# Initial Characterization of WDR5B Reveals a Role in the Proliferation of Retinal Pigment Epithelial Cells

**DOI:** 10.3390/cells13141189

**Published:** 2024-07-13

**Authors:** Jeffrey K. Bailey, Dzwokai Ma, Dennis O. Clegg

**Affiliations:** 1 Department of Molecular, Cellular and Developmental Biology, Neuroscience Research Institute, University of California, Santa Barbara, CA 93106, USA; 2 Center for Stem Cell Biology and Engineering, University of California, Santa Barbara, CA 93106, USA

**Keywords:** WDR5B, WDR5, CRISPR-Cas9, CUT&RUN, RPE, EMT, chromatin

## Abstract

The chromatin-associated protein WDR5 has been widely studied due to its role in histone modification and its potential as a pharmacological target for the treatment of cancer. In humans, the protein with highest sequence homology to WDR5 is encoded by the retrogene WDR5B, which remains unexplored. Here, we used CRISPR-Cas9 genome editing to generate WDR5B knockout and WDR5B-FLAG knock-in cell lines for further characterization. In contrast to WDR5, WDR5B exhibits low expression in pluripotent cells and is upregulated upon neural differentiation. Loss or shRNA depletion of WDR5B impairs cell growth and increases the fraction of non-viable cells in proliferating retinal pigment epithelial (RPE) cultures. CUT&RUN chromatin profiling in RPE and neural progenitors indicates minimal WDR5B enrichment at established WDR5 binding sites. These results suggest that WDR5 and WDR5B exhibit several divergent biological properties despite sharing a high degree of sequence homology.

## 1. Introduction

Eukaryotic genomic DNA is complexed with histones and additional non-histone proteins collectively known as chromatin [1]. Chromatin structure and organization can dramatically affect gene expression [2], genomic integrity [3], and cellular identity [4,5]. Histones carry dynamic post-translational modifications that actively contribute to chromatin packing density [6]. Notably, methylation of histone H3 at lysine 4 (H3K4) is enriched near the transcriptional start sites (TSS) of actively transcribed genes [7,8]. In mammals, H3K4 is methylated by MLL/SET methyltransferase complexes, which contain one catalytic subunit (SET1A, SET1B, MLL1, MLL2, MLL3, or MLL4) and four regulatory subunits required for efficient enzymatic function: WDR5, RBBP5, ASH2L, and DPY30 [9,10,11,12,13,14].

WDR5 also participates in a variety of noncanonical functions. For example, within the nucleus, WDR5 interacts with histone acetyltransferases [15,16]; histone deacetylases [17,18]; the transcription factors MYC, POU5F1, C/EBPα, HSF2, and TWIST [5,19,20,21,22]; the chromatin remodeling proteins CHD8 and INO80 [23,24,25]; and thousands of lncRNAs including HOTTIP, NEAT1, and NeST [22,26,27,28,29]. Moreover, cytoplasmic interacting partners of WDR5 include microtubule-binding proteins KIF2A and CYK4/MKLP1 [30,31], proteins from measles virus and viral response factors [32,33], the Golgi-localized receptor PAQR3 [34], as well as actin and/or γ-tubulin at the base of cilia [35,36].

Despite decades of interest in WDR5, no study to our knowledge has focused on the conserved retrogene WDR5B. Screening efforts have observed binding of WDR5B to the CUL4-DDB1 DNA damage repair complex [37] as well as the lysosomal protein PARK9 [38], although the significance of both interactions is unknown. In this study, we report that WDR5B localizes to the nucleus and, in contrast to WDR5, exhibits elevated expression upon neural differentiation. We also investigate the consequences of WDR5B depletion in stem cell-derived retinal pigment epithelial cells (hESC-RPE), a differentiated cell type of neuroectodermal origin with high relevance for the treatment of retinal diseases such as age-related macular degeneration (AMD) and proliferative vitreoretinopathy (PVR) [39,40,41]. Depletion of WDR5B in multiple, independent RPE cell lines impairs proliferation and decreases viability. Substantial differences in the chromatin binding characteristics of WDR5 and WDR5B are also apparent, indicating that WDR5 and WDR5B may perform distinct functions, despite their close homology.

## 2. Materials and Methods

### 2.1. Cell Culture

Cell culture incubators were maintained at 37 °C with 5% CO_2_. Human embryonic stem cell lines H9 (WA09, WiCell Research Institute, Madison, WI, USA) and UCSF4 (NIH registry No. 0044, University of California, Oakland, CA, USA) as well as induced pluripotent cell lines DF19-9-11T.H (WiCell Research Institute) and MyCell iPSCs (No. 1013.201, Cellular Dynamics International MyCell iPSC Services, Madison, WI, USA) were cultured in mTeSR Plus (Stem Cell Technologies, Vancouver, BC, Canada) on hESC-qualified Geltrex substrate (Thermo Fisher Scientific, Waltham, MA, USA) or hESC-qualified Matrigel (Corning, Corning, NY, USA), with media changes every other day. Stem cell lines were passaged using Versene or TrypLE Select (Thermo Fisher Scientific) with 10 µM Y27632 supplementation (Tocris Bioscience, Minneapolis, MN, USA) for one day following each passage. ARPE-19 (CRL-2302, ATCC, Manassas, VA, USA) and HEK293T (CRL-3216, ATCC) were maintained in DMEM supplemented with 10% fetal bovine serum (Atlas Biologicals, Fort Collins, CO, USA), 1× sodium pyruvate, and 1× Penicillin-Streptomycin (Thermo Fisher Scientific) and were passaged using 0.25% Trypsin (Cytiva, Marlborough, MA, USA). Stem cell-derived RPE lines were cultured in X-VIVO 10 medium (Lonza, Walkersville, MD, USA) following initial differentiation and passaged using TrypLE Select with 10 µM Y27632 following passage as previously described [42,43,44]. Other cell lines including H9T (HTB-176, ATCC), Hs27 (CRL-1634, ATCC), U87MG (HTB-14, ATCC), HeLa (CCL-2, ATCC), SHSY5Y (CRL-2266, ATCC), and HT-1080 (CCL-121, ATCC) were maintained according to ATCC recommendations. Viable cell counts were performed using a NucleoCounter NC-200 with Via-2 cassettes (Chemometec, Allerød, Denmark). All cell lines tested negative for mycoplasma contamination.

### 2.2. Antibodies

Primary antibodies used in this study were the following: rabbit-anti-WDR5 (A302-429A, Bethyl, Montgomery, TX, USA, C&R: 1:100), rabbit-anti-FLAG (14793, Cell Signaling Technology, Danvers, MA, USA, C&R: 1:100), rabbit IgG isotype control (66362, Cell Signaling Technology, C&R: 1:25), mouse-anti-FLAG (660083IG, Proteintech, Rosemont, IL, USA, ICC: 1:200, WB: 1:1000), mouse-anti-GAPDH (sc-32233, Santa Cruz Biotechnology, Dallas, TX, USA, WB: 1:1000), rabbit-anti-β-tubulin (100941AP, Proteintech, WB: 1:2000), mouse-anti-WDR5 (sc-393080, Santa Cruz Biotechnology, WB: 1:500), and rabbit-anti-HA (3724, Cell Signaling Technology, WB: 1:1000). Secondary antibodies used in this study were the following: donkey-anti-mouse IgG IRDye 680RD (926-68072, LI-COR Biosciences, Lincoln, NE, USA, WB: 1:8000), donkey-anti-mouse IgG IRDye 800CW (926-32212, LI-COR Biosciences, WB: 1:8000), donkey-anti-rabbit IgG IRDye 680RD (926-68073, LI-COR Biosciences, WB: 1:8000), donkey-anti-rabbit IgG IRDye 800CW (926-32213, LI-COR Biosciences, WB: 1:8000), and goat-anti-mouse IgG Alexa Fluor 594 Tyramide SuperBoost Kit (B40942, Thermo Fisher Scientific, ICC: pre-diluted).

### 2.3. Neural Ectoderm Differentiation

hPSCs were lifted using TrypLE Select and seeded at 50,000 cells/cm^2^ in mTeSR Plus with 10 µM Y27632 on hESC-qualified Geltrex. One day after seeding, Y27632 was removed and cells were recovered for an additional two days in mTeSR Plus. Differentiation was initiated using a neural induction media composed of DMEM/F12, 1× B-27, 1× N-2, 10 mM nicotinamide (Sigma Aldrich, St. Louis, MO, USA), 10 µM SB431542 (1614, Tocris Bioscience), and 5 µM DMH1 (4126, Tocris Bioscience). Media changes were performed every two days and cells were harvested on day 6 or as indicated.

### 2.4. Cell Proliferation and Viability Assays

ARPE-19 and hESC-RPE were seeded at 17,000 cells/cm^2^ and 20,000 cells/cm^2^, respectively, in 48-well tissue culture plates. For ARPE-19 proliferation assays, cells were seeded in DMEM with 10% FBS, 1× sodium pyruvate, 1× penicillin-streptomycin and transitioned at one day post-seeding to X-VIVO 10 medium. For hESC-RPE proliferation assays, cells were seeded on hESC-qualified Geltrex-coated 48-well plates in X-VIVO 10 medium with 10 µM Y27632. After 5 days, Y27632 was removed and cells were maintained in X-VIVO 10. At the indicated timepoints post-seeding, cultures were stained using X-VIVO 10 supplemented with Hoechst 33342 (H3570, Thermo Fisher Scientific, 1:1000) and propidium iodide (P3566, Thermo Fisher Scientific, 1:1000) followed by a 20 min incubation at 37 °C with 5% CO_2_. Total nuclei (Hoechst-positive) and dead nuclei (propidium iodide-positive) were counted using a Celigo Image Cytometer (200-BFFL-S, Nexcelom Bioscience, Lawrence, MA, USA). For each cell line, at least three independently seeded experiments were performed.

### 2.5. Cloning, Production of Lentiviral Particles, and Stable Line Generation

For WDR5-FLAG and WDR5B-3XFLAG overexpression, the pLVXpuro-P2A-T2A lentivector was first generated from pLVXpuro (Clontech/Takara Bio, Mountain View, CA, USA) by replacing the sequence between the XhoI and AgeI sites with a P2A-T2A gene fragment as indicated in Table S1 (Twist Biosciences, South San Francisco, CA, USA). Human WDR5B-3XFLAG was amplified from the gDNA of WDR5B-3XFLAG knock-in H9 ESCs and cloned into pEGFP-C1 between AgeI and EcoRI sites for initial sequence verification. For production of lentiviral particles, WDR5B-3XFLAG was amplified from the pEGFP-C1 template and cloned into the pLVXpuro-P2A-T2A lentivector between XhoI and XbaI sites. Human WDR5-FLAG was synthesized as a gene fragment (Table S1, Twist Biosciences) and cloned into pLVXpuro-P2A-T2A between XhoI and NotI sites. All inserts were verified by Sanger sequencing. Bacterial glycerol stocks of shRNA constructs in the pLKO.1 lentivector were purchased from Sigma Aldrich, and sequence details are listed in Table S1. To produce lentiviral particles, HEK293T cells were seeded at approximately 40,000 cells/cm^2^ in 10 cm cell culture dishes two days before transfection. Each 10 cm dish was transfected with a mixture containing 3 µg of lentivector, second generation packaging plasmids (1.96 µg psPAX2 and 1.06 µg pMD2.G [Addgene, Watertown, MA, USA]), and 1:50 Turbofect transfection reagent (R0531, Thermo Fisher Scientific) diluted in Opti-MEM. At 16–18 h post-transfection, a medium exchange was performed and supernatant containing lentiviral particles was collected after incubation for an additional 24 h. Supernatant was 0.22 µm filtered, concentrated using PEG-it (LV810A-1, System Biosciences, Palo Alto, CA, USA) according to the manufacturer’s instructions, and stored in aliquots at −80 °C prior to use. All lentivirus transductions were performed in media containing 10 µg/mL polybrene (TR-1003, Sigma Aldrich) for 5–7 h, and medium for selection was supplemented with 3 µg/mL puromycin dihydrochloride (A1113803, Thermo Fisher Scientific). The functional titer of each lentivirus preparation was determined by transduction of HT-1080 cells using an eight-point dilution curve followed by puromycin selection and quantification of remaining Hoechst-positive nuclei per well using a Celigo Image Cytometer. To avoid counting cells with multiple integrations, wells exhibiting > 40% survival relative to the untreated control were excluded from the titer calculation. For production of stable cell lines, ARPE-19 cells were transduced at an MOI of 2 for shRNA constructs and MOI of 6 for WDR5B-3XFLAG and WDR5-FLAG constructs. Puromycin selection was initiated at day three post-transduction.

### 2.6. Immunocytochemistry, Confocal Fluorescence Microscopy, and Image Analysis

Cells for immunocytochemistry were grown on Matrigel-coated 12 mm #1.5 pre-sterilized glass coverslips (GG121.5PRE, Neuvitro Corporation, Camas, WA, USA). Cells were fixed for 20 min at room temperature with 4% formaldehyde in 1× PBS, permeabilized for 5 min with 0.1% Triton X-100 in 1× PBS, washed three times with 1× PBS, and blocked for 30 min at room temperature with blocking buffer (1% BSA and 5% normal goat serum in 1× PBS). Primary antibodies were diluted in blocking buffer and incubated for either 2 h at room temperature or overnight at 4 °C. After three washes with 1× PBS, secondary antibodies diluted in blocking buffer were incubated for 1 h at room temperature. For endogenous WDR5B-3XFLAG staining, enzymatic amplification of secondary antibody signal was performed using the Alexa Fluor 594 Tyramide SuperBoost Kit according to the manufacturer’s instructions (B40942, Thermo Fisher Scientific). Coverslips were subsequently incubated for 10 min with 1:1000 Hoechst 33342 in 1× PBS, washed three times with 1× PBS, and mounted in ProLong Gold Antifade (Thermo Fisher Scientific). Image acquisition was performed using an Olympus FLV1000S spectral laser scanning confocal, a PlanApo N 60XOSC objective, and excitation laser lines at 405, 488, 559, and 635 nm (Olympus Life Sciences, Shinjuku-ku, Tokyo, Japan). Image analysis was performed using ImageJ-FIJI (National Institutes of Health, Bethesda, MD, USA). For nearest neighbor analysis of cell density from 10× phase contrast images, cell segmentation by pixel classification was performed using Ilastik 1.4.0 [45] followed by first nearest neighbor distance measurement with CellProfiler 4.2.6 [46] using the ‘MeasureObjectNeighbors’ module. Quantification was performed on 6 fields of view per ARPE-19 cell line (2664 ± 577 cells per field) and 3 fields of view per hESC-RPE cell line (2391 ± 741 cells per field).

### 2.7. CRISPR-Cas9 Genome Editing

Both WDR5B knockout ESCs and 3XFLAG-tagged WDR5B knock-in ESCs were derived from the H9 stem cell line (WA09, WiCell Research Institute). Guide RNAs (designed as crRNAs), Alt-R S.p. Cas9 Nuclease V3 (Cas9), single-stranded DNA oligonucleotide homology directed repair (ssODN-HDR) templates, and genotyping primer–probe sets were purchased from Integrated DNA Technologies (Coralville, IA, USA), and sequence details are available in Table S1. Knockout of WDR5B was performed by removal of the entire coding sequence using a pair of WDR5B-flanking guide RNAs. Dual-guide RNA strategies have been successfully used to remove genomic regions up to 8 kb in length with high efficiency [47]. Knock-in of a 3XFLAG tag at the C-terminus of endogenous WDR5B was performed using an ssODN-HDR template and a single guide RNA (Table S1). To anneal guide RNA oligonucleotides, crRNA and tracrRNA were mixed at a concentration of 50 µM each in nuclease-free IDTE, pH 7.5, heated to 95 °C for 5 min, and slowly cooled to room temperature. Ribonucleoprotein complexes (RNPs) of approximately 20 µM Cas9:crRNA:tracrRNA were assembled by mixing Cas9 enzyme and crRNA:tracrRNA duplex in 1× PBS followed by room-temperature incubation for 5 min. Immediately prior to nucleofection, RNPs were diluted to a final concentration of approximately 2 µM in nucleofection solution (VPH-5012, Lonza) with 2 µM ssODN-HDR template added for knock-in lines. ESCs were lifted with TrypLE Select (Thermo Fisher Scientific) and 1 × 10^6^ cells were washed once with 1× PBS, resuspended in nucleofection solution with RNP mixture, electroporated using a Lonza Nucleofector IIB set to program ‘A-32’, and seeded onto Matrigel-coated tissue culture plates in mTeSR Plus supplemented with 10 µM Y27632. Once ESCs regained good colony morphology, clonal lines were established by limiting dilution in Matrigel-coated 96-well plates by seeding cells at a target density of 1 cell per well in mTeSR Plus with 1× CloneR (05888, Stem Cell Technologies). Media changes were performed according to the manufacturer’s protocol. After 2–3 weeks in culture, many colonies reached >30% confluence and were lifted with Versene for expansion into 24-well plates. For each colony, 80% of the cell suspension was used for cell line propagation and 20% was lysed for qPCR genotyping in 0.5× Direct-Lyse buffer (10 mM Tris pH 8.0, 2.5 mM EDTA, 0.2 M NaCl, 0.15% SDS, 0.3% Tween-20) with thermal lysis and 2- to 10-fold dilution in water as previously described [48]. For both knock-in and knockout editing strategies, genotype screening was performed by multiplex TaqMan qPCR using custom-designed primer–probe mixes (20× stock) containing 3 µM HEX-labeled wild-type WDR5B probe, 3 µM FAM-labeled probe for the edited allele, and 10 µM each of two (knock-in) or three (knockout) unlabeled primers to facilitate amplification. To confirm qPCR genotyping results, genomic DNA was extracted using the Qiagen DNeasy Kit (69506, Qiagen, Hilden, Germany) from several clones per genotype (wild-type, WDR5B knockout, and WDR5B-3XFLAG knock-in). Approximately 1.4 kb of genomic sequence surrounding the WDR5B genomic locus was amplified from each clonal line using primers F2 and R3 (Table S1) and cloned into the pEGFP-C1 plasmid between the AgeI and EcoRI sites. Inserts were confirmed by Sanger sequencing (Azenta Life Sciences, Burlington, MA, USA and Eton Bioscience, San Diego, CA, USA). PCR amplification was performed using TaqMan Gene Expression Master Mix for qPCR genotyping (4369016, Thermo Fisher Scientific), GoTaq Flexi for agarose gel electrophoresis (M8295, Promega, Madison, WI, USA), or Phusion polymerase for cloning (M0530S, New England Biolabs, Ipswich, MA, USA).

### 2.8. SDS-PAGE and Western Blotting

Lysates were collected by lifting cells using TrypLE Select (Thermo Fisher Scientific) and quenching with growth media followed by one wash with 1× PBS. Pelleted cells were resuspended in ice cold RIPA buffer (PI89900, Thermo Fisher Scientific) supplemented with 1× protease inhibitor (1862209, Thermo Fisher Scientific) and 1× phosphatase inhibitor (1862495, Thermo Fisher Scientific). Lysates were mixed end-over-end at 4 °C followed by clearing centrifugation for 10 min at 16,000× *g* and 4 °C. Supernatants were transferred to fresh tubes on ice, and protein concentrations were quantified using the Pierce BCA assay (23227, Thermo Fisher Scientific). Samples were denatured with 4× NuPage LDS Sample Buffer (NP0008, Thermo Fisher Scientific) supplemented with 3.125% β-mercaptoethanol (Sigma Aldrich) and heated to 85 °C for 15 min. Equal amounts of total protein per sample were loaded into 4–12% Bis-Tris precast gels (Thermo Fisher Scientific) and proteins were separated in 1× MOPS buffer (Thermo Fisher Scientific) at 100 V. After electrophoresis, proteins were transferred to 0.45 µm PVDF membranes (Millipore-Sigma, Burlington, MA, USA) using a Bio-Rad wet transfer apparatus and Towbin buffer (25 mM Tris, 192 mM glycine, 20% *v*/*v* methanol) for 60 min at 80 V with ice cooling. Membranes were sequentially rinsed in water, methanol, and dried completely at 37 °C before probing with primary antibodies diluted in blocking buffer, which consisted of a 1:1 mixture of fluorescence blocking buffer (MB-070, Rockland Immunochemicals, Limerick, PA, USA) and PBST (1× PBS with 0.1% Tween-20). Primary antibody incubations were conducted overnight at 4 °C with gentle agitation. Following primary incubations, membranes were washed three times with PBST for 10 min each followed by incubation with IRDye 680 nm or 800 nm antibodies (LI-COR Biosciences) for 45 min at room temperature in blocking buffer. After three additional PBST washes, membranes were dried completely, imaged on an Odyssey Imaging System (LI-COR Biosciences), and quantification was performed using Odyssey V3.0 software.

### 2.9. RNA Purification and Quantitative Real-Time PCR (RT-qPCR)

Cells were lysed in RLT buffer (Qiagen) and stored at −80 °C prior to purification. Total RNA was isolated using the RNeasy Mini kit with on-column DNase digestion of genomic DNA according to the manufacturer’s instructions (Qiagen). RNA yields were quantified by 260 nm absorbance measurement using a SynergyH1 plate reader (BioTek, Winooski, VT, USA), and 25 ng of RNA per well was used for RT-qPCR. Reverse transcription and PCR amplification were performed using EXPRESS One-Step SYBR GreenER master mix (11780200, Thermo Fisher Scientific) for SYBR green RT-qPCR or AgPath-ID One-Step RT-PCR Reagents (4387391, Thermo Fisher Scientific) for TaqMan RT-qPCR on a CFX96 Real-Time detection instrument (BioRad, Hercules, CA, USA). Linearized data for each sample were first normalized to the geometric mean of reference genes EIF2B2 and UBE2R2, and any subsequent normalization to control samples was performed as indicated. For CUT&RUN-qPCR, equal sample volumes per well of CUT&RUN DNA were loaded except for input only samples, which were diluted 10-fold prior to loading. CUT&RUN-qPCR was performed using PowerUp SYBR Green Master Mix for qPCR (A25742, Thermo Fisher Scientific). All primer sequences are listed in Table S1.

### 2.10. RT-qPCR Array for Apoptosis-Related Gene Expression

Total RNA was purified from ARPE-19 stable lines (control shRNA, WDR5B shRNA #1, WDR5B shRNA #2) on day 8 post-seeding as described above and reverse transcribed to cDNA using iScript Reverse Transcription Supermix (1708841, Bio-Rad). Duplicate Human Cellular Apoptosis Pathway qPCR array plates (4414117, Thermo Fisher Scientific) for each stable line were loaded with cDNA, and qPCR was performed using TaqMan Gene Expression Master Mix (4369016, Thermo Fisher Scientific) on a BioRad CFX96 Real-Time detection instrument. Linearized gene expression values were normalized to the geometric mean of reference genes GAPDH and GUSB, and genes on the array plate that were not detected (Ct > 40) or that exhibited high variability between replicates (CV > 70%) were excluded from further analysis. Expression data for each WDR5B shRNA line were normalized to the control shRNA line and represented as a heatmap of log2-transformed fold changes sorted by the mean of both values.

### 2.11. CUT&RUN

CUT&RUN was performed using a similar protocol to those previously described [49,50]. Immediately prior to lifting the cells, magnetic concanavalin A (Con A) beads (BP531, Bangs Laboratories, Fishers, IN, USA) were activated by rinsing twice in cold binding buffer (20 mM HEPES pH 7.5, 10 mM KCl, 1 mM CaCl_2_, 1 mM MnCl_2_) and stored on ice in binding buffer equivalent to the original bead volume. Con A beads were always mixed by gentle pipetting or flick mixing rather than by vortexing. Cells were rinsed with 1× PBS, lifted with 0.25% Trypsin, and quenched with 2–3 volumes of growth media. Cell count and viability were determined using a NucleoCounter NC-200, and 3 × 10^6^ cells per sample (ARPE-19) to 6 × 10^6^ cells per sample (neural ectoderm) were pelleted, washed once with 1× PBS, washed once with wash buffer (20 mM HEPES pH 7.5 and 150 mM NaCl supplemented with 1:100 spermidine [27287, Cell Signaling Technology] and 1:200 protease inhibitor cocktail [7012, Cell Signaling Technology]), and resuspended in wash buffer of the same composition. Activated Con A beads were added to the cell suspension, and binding was achieved by end-over-end mixing at room temperature for 15 min. Cells bound to Con A beads were resuspended in antibody buffer (wash buffer supplemented with 2 mM EDTA, and 0.01% digitonin [300410, Millipore-Sigma]) and aliquoted into equal portions including an input-only control. Antibodies were added as detailed above, and incubations were conducted at 4 °C overnight. Following antibody binding, beads were washed twice with wash buffer supplemented with 0.01% digitonin and incubated for 10 min at room temperature with 1:25 CUTANA pAG-MNase for CUT&RUN (15-1016, EpiCypher, Durham, NC, USA) diluted in wash buffer of the same composition. Beads were washed twice with wash buffer containing 0.01% digitonin, and pAG-MNase digestion was performed at 4 °C for 2 h in wash buffer supplemented with 0.01% digitonin and 2 mM CaCl_2_. Digestion was stopped by the addition of 2× stop buffer (340 mM NaCl, 20 mm EDTA, 4 mm EGTA) supplemented with 0.01% digitonin, 1:200 of 10 mg/mL RNAse A (7013, Cell Signaling Technology), and 50 pg per sample of *S. cerevisiae* spike-in normalization DNA (36598S, Cell Signaling Technology). Samples were incubated at 37 °C for 15 min with gentle agitation to release DNA, supernatant was separated from magnetic Con A beads into a fresh tube, and samples were diluted with an equal volume of 0.1× TE buffer (1 mM Tris-HCl pH 7.5, 0.1 mM EDTA). Samples were stored at −20 °C prior to DNA purification.

To process input-only samples, cells were bound to Con A beads and incubated overnight in antibody buffer as described above. Input samples were subsequently resuspended in buffer A (15 mM Tris-HCl pH 7.5, 60 mM KCl, 15 mM NaCl, 5 mM MgCl_2_, 300 mM sucrose, 0.25% NP-40, and supplemented on the day of use with 1 mM DTT and protease inhibitor cocktail) and were incubated on ice for 12 min with brief mixing by inversion every 3 min. Samples were washed once with buffer B (50 mM Tris-HCl pH 7.5, 4 mM MgCl_2_, 1 mM CaCl_2_, 320 mM sucrose, and supplemented on the day of use with 1 mM DTT and protease inhibitor cocktail) and resuspended in one original sample volume of buffer B. Micrococcal nuclease (10011, Cell Signaling Technology) was diluted 1:40 in buffer B and added to each input sample at 1:100 (final dilution 1:4000), and samples were digested at 37 °C for 15 min with gentle agitation. To stop digestion, samples were supplemented with 1:10 of 0.5 M EDTA, 1:50 of 20 mg/mL proteinase K (10012, Cell Signaling Technology), 1:100 of 10% SDS, and 1:200 of 10 mg/mL RNAse A. Samples were incubated at 65 °C for 2 h, supernatant was separated from magnetic Con A beads into a fresh tube, and samples were diluted with an equal volume of 0.1× TE buffer. Samples were stored at −20 °C prior to DNA purification.

DNA purification of input and CUT&RUN samples was performed by Phenol:Chloroform:Isoamyl Alcohol extraction as previously described [49]. Briefly, samples were thawed, supplemented with 1:100 of 10% SDS, 1:100 of 20 mg/mL proteinase K, and incubated for 10 min at 70 °C. One sample volume of UltraPure Phenol:Chloroform:Isoamyl Alcohol 25:24:1 *v*/*v* (15593031, Thermo Fisher Scientific) was added and samples were mixed by full-speed vortexing, transferred to phase-lock tubes (129046, Qiagen), and centrifuged at room temperature for 5 min at 16,000× *g*. One sample volume of chloroform was added, mixed by inversion, and samples were centrifuged at room temperature for 5 min at 16,000× *g*. The aqueous upper phase was removed into a fresh microfuge tube containing 4 µg glycogen (10901393001, Millipore-Sigma), 2.5 volumes of ice cold 100% ethanol were added, and samples were mixed by vortexing followed by incubation on ice for 10 min. Samples were centrifuged at 4 °C for 10 min at 16,000× *g*, supernatant was gently discarded, samples were washed with 5 volumes of ice cold 100% ethanol, centrifuged at 4 °C for 2 min at 16,000× *g*, supernatant was gently discarded, and pellets were allowed to air dry for 5–10 min. After excess liquid had evaporated, pellets were resuspended in 0.1× TE buffer, transferred to fresh tubes, and stored at −20 °C. Libraries were constructed by DNA end repair, A-tailing, Illumina adapter ligation, size selection, PCR amplification, and quantification by qPCR. Both library construction and 150 bp paired-end sequencing on an Illumina NovaSeq 6000 were performed by Novogene Corporation Inc. (Beijing, China). Two independent CUT&RUN experiments were performed and sequenced for each condition.

### 2.12. CUT&RUN and ChIP-Seq Analysis

Previously published WDR5 ChIP-seq data from Be2C cells (GEO: GSE136451) and WDR5 CUT&RUN data from HeLa S3 cells (GEO: GSE185921) were downloaded from the NCBI Sequence Read Archive and extracted to compressed FASTQ files using fastq-dump 3.0.2 with the parameters: ‘--gzip --skip-technical --readids --read-filter pass --dumpbase --split-3 --clip’. Initial analysis steps including read quality control and adapter trimming (trimgalore 0.6.7, fastqc 0.11.9, cutadapt 3.4), alignment to hg38 (bwa-mem 0.7.17), marking and filtering out duplicate reads, filtering out reads in blacklisted regions (hg38-blacklist.v2.bed) [51], and filtering out improperly mapped reads (picard 2.27.4, samtools 1.15.1, bedtools 2.30.0) were performed using the nf-core/chipseq Nextflow pipeline v2.0.0 with default parameters [52]. Peak calling was performed using MACS3 3.0.0b1 with thresholds of q ≤ 0.05 and *p* ≤ 0.001 as indicated. Peak set overlap analysis was conducted using DiffBind 3.8.4 [DOI: 10.18129/B9.bioc.DiffBind] [53], and peak annotation of promoters was performed using ChIPSeeker 1.34.1 [DOI: 10.18129/B9.bioc.ChIPseeker] [54]. DiffBind consensus peaks were defined as sites found in all biological replicates for a given cell line. BigWig files used to produce heatmaps were generated with deeptools 3.5.1 bamCompare and the parameters ‘-bs 10 --smoothLength 30 --extendReads 150 --centerReads --scaleFactorsMethod None --normalizeUsing BPM’, and heatmaps were plotted using deeptools computeMatrix and plotHeatmap. BigWig files for peak visualization with the Integrative Genomics Viewer (IGV) were also produced with bamCompare using BPM normalization only. CUT&RUN sequencing data from this study were deposited in the NCBI GEO repository with the accession number GSE244882.

### 2.13. In Vitro Nucleosome Binding Assay

The nanoluciferase-HA tag was amplified from a gene synthesis fragment (Table S1, Twist Biosciences) and cloned between NdeI and BlpI sites of the PURExpress Control DHFR Plasmid (N0424AVIAL, New England Biolabs) using primers as indicated in Table S1. WDR5-NLUC-HA and WDR5B-NLUC-HA coding sequences were cloned between XbaI and BamHI sites of the PURExpress Control DHFR Plasmid using a three-fragment ligation after amplification from pLVXpuro-WDR5-FLAG, pLVXpuro-WDR5B-3XFLAG, and NLUC-HA templates with the primers listed in Table S1. In vitro transcription and translation were performed for 4 h at 37 °C using the PURExpress Protein Synthesis Kit (E6800S, New England Biolabs) with 350 ng of template plasmid per reaction and supplemented with RNaseOUT. Reactions were subsequently diluted 20-fold in Pierce IP buffer (Thermo Fisher Scientific) on ice. NLUC-HA tagged proteins were bound to Pierce anti-HA magnetic beads (88836, Thermo Fisher Scientific) for 1 h at 4 °C, washed three times with Pierce IP buffer, and resuspended in approximately two bead volumes of Pierce IP buffer. Activity of nanoluciferase on the beads as well as Western blotting were used to confirm successful protein synthesis and affinity purification. For nucleosome binding assays, equivalent bead volumes for each construct were washed three times in CUT&RUN antibody buffer (20 mM HEPES pH 7.5, 150 mM NaCl, 0.5 mM spermidine, 2 mM EDTA, 0.01% digitonin) and incubated in CUT&RUN antibody buffer supplemented 1:50 with SNAP-ChIP K-MetStat recombinant nucleosomes (19-1001, EpiCypher) for 1 h at 4 °C. Beads were then washed three times in CUT&RUN antibody buffer and bound nucleosome DNA was recovered using the GeneJET Gel Extraction Kit (Thermo Fisher Scientific). Quantification of nucleosome DNA was performed by qPCR using the primer–probe set indicated in Table S1.

### 2.14. Visualization of Homology and Gene Collinearity

To visualize gene collinearity, genome-wide RefSeq annotations (in GFF format) and protein sequences (in FASTA format) were obtained for human (GRCh38.p14), Rhesus macaque (Mmul10), mouse (GRCm39), and chicken (GRCg6a) from the NCBI genomes database. GFF and FASTA files were parsed using the Python libraries gffpandas 1.2.0 and biopython 1.79, respectively. All loci annotated as ‘CDS’ (protein coding sequences) within 200 kb upstream or downstream of the WDR5B locus (or WDR5B-flanking genes in the case of chicken) were extracted and concatenated into multi-FASTA files for pairwise species comparisons. All-by-all BLAST was performed using the NCBI ‘blastall’ command line tool with the options: ‘-p blastp -e 1e-10 -b 5 -v 5 -m 8’. The BLAST output and corresponding GFF coordinate annotations were used to construct gene collinearity maps with MCScanX via the TBtools wrapper [55,56]. Collinearity diagrams were generated from the MCScanX output using SynVisio [57] with manual annotations added using Adobe Illustrator. To construct Figure S1B, RefSeq protein sequences corresponding to six human genes (WDR5, WDR5B, as well as the immediately upstream and downstream genes at each locus: FAM162A, KPNA1, BRD3, and RXRA) were used to perform standard protein–protein BLAST (blastp) searches against NCBI databases of all annotated proteins for each listed organism. Results were saved in single-file JSON format and parsed with the biopython 1.7.9 SeqIO and Entrez modules to filter up to 40 BLAST hits with the lowest E-values (highest homology) for each query and to verify that these hits appropriately corresponded to adjacent genes on the same chromosome or genomic scaffold. For organisms with no WDR5B BLAST hits located between the FAM162A and KPNA1 genes, the absence of an annotated protein at this genomic region was manually verified using NCBI Genome Browser. All diagrams depicting chromosomal locations and gene structures were adapted from Ensembl (release 109). Sequence differences between WDR5 and WDR5B as well as residues known to be involved in protein–protein interactions (WIN residues: Ala65, Ser91, Asp107, Phe133, Tyr191, Tyr260, Phe263; WBM residues: Asn225, Tyr228, Leu240, Phe266, Val268, Gln289) were depicted on the structure of WDR5 (PDB ID: 2H14) using UCSF Chimera.

### 2.15. Statistical Analysis

Unless otherwise specified, all statistical analyses were performed as follows using GraphPad Prism v6.01. For statistical analysis of two groups, two-tailed unpaired *t*-tests were performed. Statistical analysis of more than two groups was conducted by univariate ANOVA with Tukey’s HSD post hoc. Asterisks indicate statistical significance as follows: **** (*p* < 0.0001), *** (*p* < 0.001), ** (*p* < 0.01), * (*p* < 0.05), ns (not significant). Summary plots represent mean ± standard deviation error bars and biological replicates are plotted.

## 3. Results

### 3.1. WDR5B Is Conserved in Eutherian Mammals and Expressed in Human Cells

**Figure 1:**
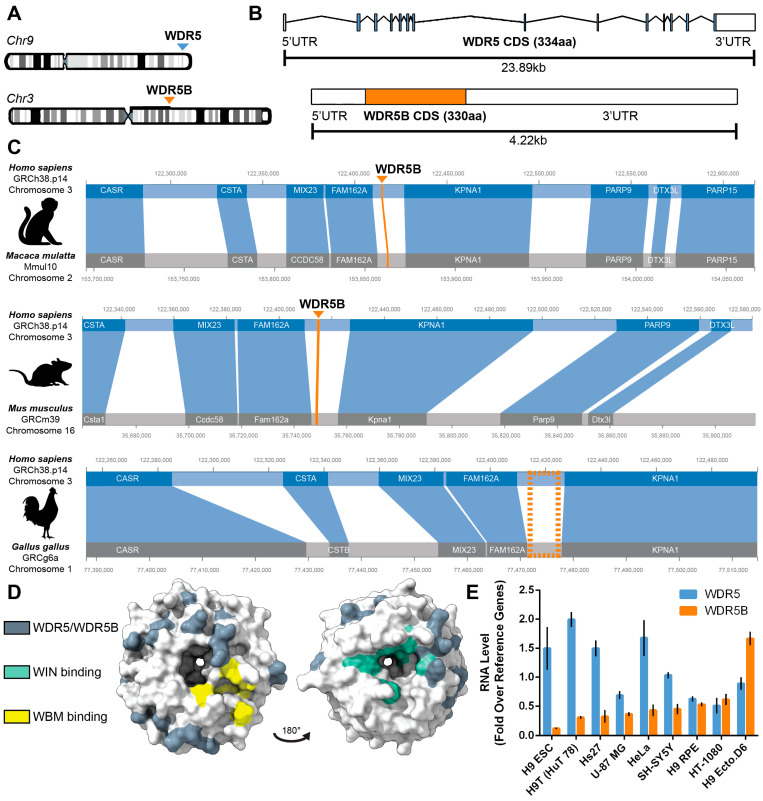
Mammalian genomes encode and express WDR5B. (**A**) Human WDR5 and WDR5B are located on chromosomes 9 and 3, respectively. (**B**) Human WDR5 contains 14 exons, while human WDR5B encodes an intronless transcript. Shaded regions indicate the protein coding sequences (CDS). (**C**) Gene collinearity surrounding WDR5B is conserved among vertebrates, but non-mammalian vertebrates such as chicken lack WDR5B. Blue-shaded regions indicate homologous genes, and the dotted orange rectangle marks the location where a WDR5B homolog would be expected in chicken but was not found. (**D**) Visualization of differences between WDR5 and WDR5B (dark gray, conservative substitutions included) on the structure of WDR5 (PDB ID: 2H14). Residues required for protein–protein interactions at the WBM and WIN binding pockets of WDR5 (yellow and green, respectively) are conserved in WDR5B. Unstructured N-termini of WDR5 and WDR5B are not depicted. (**E**) RNA levels of WDR5 and WDR5B are inversely correlated in nine human cell lines. Spearman coefficient: −0.70 (*p* = 0.043).

To determine if WDR5B might encode a functional protein, we first compared its gene structure, evolutionary conservation, and mRNA expression profile to that of WDR5. In humans, WDR5 and WDR5B are located on chromosomes 9 and 3, respectively (Figure 1A). Similar to other retrogenes [58], WDR5B encodes an intronless transcript while WDR5 exhibits exon–intron gene structure (Figure 1B). Since WDR5 is conserved from yeast to humans [9], we wondered if WDR5B orthologs also exist. Among the 40 vertebrate genomes we examined, separate genes encoding WDR5 and WDR5B were only found in eutherian mammals (Figure S1). In contrast, monotreme, marsupial, and non-mammalian vertebrate genomes appear to encode a single gene with high homology to WDR5 and which exhibits exon–intron gene structure. Gene collinearity analysis of a ~400 kb region surrounding human WDR5B showed a conserved arrangement of upstream and downstream genes flanking WDR5B in macaque, mouse, and chicken despite the absence of WDR5B in chicken (Figure 1C). Similarly, a conserved order of upstream and downstream genes at both the WDR5 and WDR5B loci was confirmed in all 40 vertebrate genomes regardless of the presence or absence of WDR5B (Figure S1). Together these data suggest that insertion of the WDR5B retrogene may be unique to eutherian mammals.

To the best of our knowledge, no studies have directly investigated whether the WDR5B locus yields a protein product, and no WDR5B antibodies have been validated in published literature. The amino acid sequences of human WDR5 and WDR5B are ~86% identical with a similarity of 95% when conservative substitutions are included [59]. Residues required for protein–protein interactions at the WBM and WIN binding sites of WDR5 are conserved in WDR5B (Figure 1D). Since extensive homology might suggest functional redundancy, we were surprised to find that expression patterns of WDR5 and WDR5B in human and mouse tissues differed significantly. Using the StemMapper database of 798 mouse and 166 human transcriptomes [60], we observed that most pluripotent cell samples ranked above the 50th percentile for WDR5 expression but below the 50th percentile for expression of WDR5B (Figure S2). In contrast, differentiated cells or tissues derived from ectoderm, mesoderm, and endoderm ranked higher for WDR5B and lower for WDR5 expression. To independently assess this relationship, we compared levels of WDR5 and WDR5B by RT-qPCR in six immortalized cell lines as well as H9 human embryonic stem cells (H9 ESCs), H9-derived hESC-RPE (H9 RPE), and H9-derived neural ectoderm progenitors at day 6 of differentiation (H9 Ecto.D6). In H9 ESCs, high levels of WDR5 and low levels of WDR5B were observed, while the highest levels of WDR5B were found in neural progenitors (Figure 1E). Similar to the StemMapper data, expression of WDR5 and WDR5B was inversely correlated in these nine samples (Spearman correlation: −0.70, *p* < 0.05).

**Figure 2:**
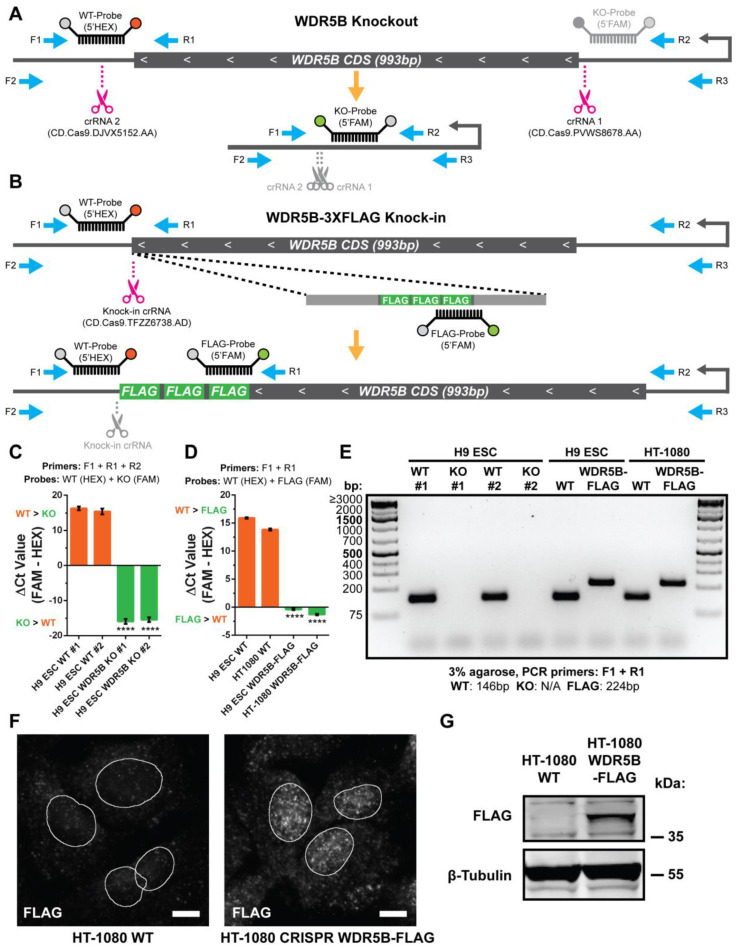
Generation of WDR5B knockout and WDR5B-FLAG knock-in cell lines. (**A**) Schematic of the human WDR5B locus with approximate locations of primers (blue arrows), CRISPR-Cas9 guide RNAs (magenta scissors), and TaqMan probes for qPCR genotyping. WDR5B is transcribed in the antisense direction (gray arrows, TSS). The orange arrow and lower diagram indicate the expected consequence of CRISPR-Cas9 editing, which disrupts the guide RNA target sequences (gray scissors). Signal from the KO probe is suppressed under wild-type conditions due to the large and inefficiently produced F1-R2 amplicon (1209 bp), short PCR extension time (30 s), and more favorable amplification conditions for the small F1-R1 WT product (146 bp). Deletion of the WDR5B coding sequence reduces the expected F1-R2 KO amplicon size to 160 bp and results in FAM signal from the cleaved KO probe while simultaneously disrupting the binding sites for the WT probe and R1 primer. (**B**) Schematic of 3XFLAG tag insertion at the C-terminus of WDR5B (top) and expected editing consequence (bottom). Signal from both the WT and FLAG probes is expected upon successful FLAG tag insertion. After editing, separation of the PAM site from the knock-in guide RNA target sequence prevents re-cutting of the edited allele (gray scissors). (**C**) Multiplex qPCR genotyping of wild-type and WDR5B-null H9 ESC clones using a mix of three primers (F1, R1, R2) and two probes (WT-HEX and KO-FAM). (**D**) Multiplex qPCR genotyping of wild-type and WDR5B-FLAG H9 ESC and HT-1080 clones using a mix of two primers (F1, R1) and two probes (WT-HEX, FLAG-FAM). For multiplex qPCR, a negative ΔCt value (FAM − HEX) indicates higher abundance of the edited vs. wild-type allele (n = 3 experimental replicates). In the case of no amplification, a Ct value of 40 was assigned. Significance was assessed by ANOVA with Tukey’s post hoc, and asterisks indicate the maximum *p*-value where both edited lines differed from both wild-type lines (**** *p* < 0.0001). (**E**) Primers F1 and R1 produced PCR products of the expected sizes (WT: 146 bp, KO: no product, FLAG: 224 bp) and indicate complete loss of WDR5B in the knockout lines. No evidence of the wild-type allele was detected in any of the edited H9 ESC or HT-1080 clonal lines. (**F**) Immunostaining confirmed nuclear localization of endogenously expressed WDR5B-FLAG in knock-in HT-1080 cells. Outlines of nuclei (white) were generated from Hoechst staining using the ImageJ Cell Magic Wand plugin. Scale bars: 10 µm. (**G**) Western blot verification of WDR5B-FLAG expression in knock-in HT-1080 cells.

Since WDR5B expression is low in pluripotent cell types, we hypothesized that stem cells might tolerate the loss of WDR5B. Conversely, WDR5 is essential for the normal viability, self-renewal, and pluripotency of mouse embryonic stem cells [5,61]. We used CRISPR-Cas9 genome editing to generate both WDR5B-knockout (Figure 2A) and WDR5B-3XFLAG epitope-tagged H9 ESCs lines (Figure 2B, hereafter referred to as WDR5B-FLAG). We also generated a WDR5B-FLAG HT-1080 line as WDR5B is elevated in HT-1080 cells (Figure 1E). The consequences of CRISPR-Cas9 editing were verified by multiplex TaqMan qPCR genotyping (Figure 2C,D), amplification of the targeted region with multiple independent sets of primers (Figure 2E and Figure S3), and Sanger sequencing (Figure S3). Immunofluorescence and Western blotting indicated a nuclear-localized protein of the predicted size in WDR5B-FLAG HT-1080 lines, although endogenous expression levels were modest (Figure 2F,G). These data reveal that human cells indeed express WDR5B mRNA and protein, and that mRNA levels of WDR5B appear to be inversely correlated with pluripotency as well as WDR5 expression.

### 3.2. Directed Neural Differentiation Upregulates WDR5B

**Figure 3:**
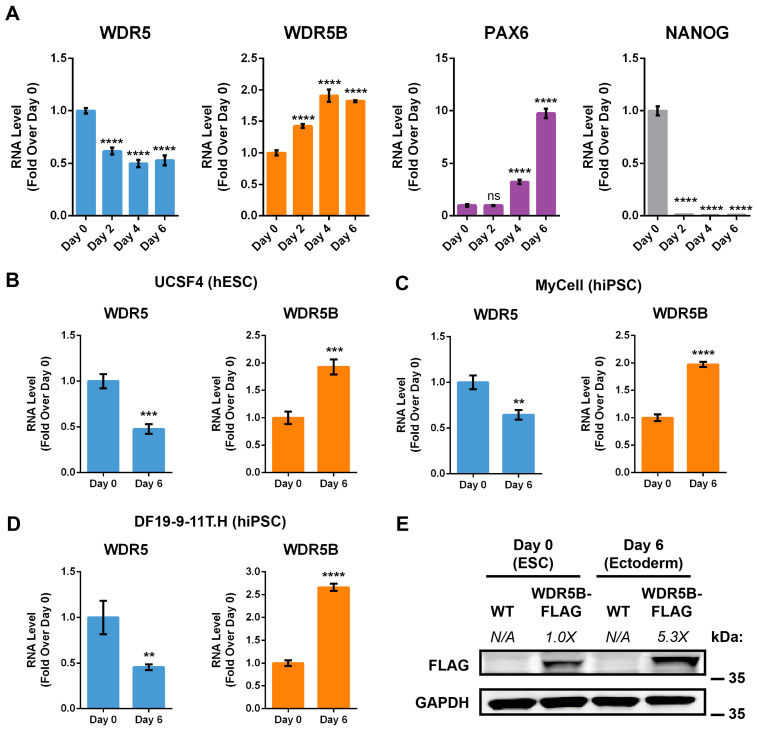
WDR5 and WDR5B exhibit opposite changes in abundance upon neural differentiation. (**A**) Neural differentiation of H9 ESCs for 6 days increased mRNA levels of WDR5B and the neural marker PAX6, while levels of WDR5 and the pluripotency marker NANOG decreased. (**B**–**D**) Neural differentiation upregulated WDR5B and downregulated WDR5 in another ESC line (UCSF4) as well as two hiPSC lines (MyCell and DF19-9-11T.H). (**E**) Six-day neural differentiation increased levels of endogenous WDR5B-FLAG 5.3-fold normalized to GAPDH. Error bars: ±SD, n = 3 experimental replicates per group. ns (not significant), ** *p* < 0.01, *** *p* < 0.001, **** *p* < 0.0001.

Since WDR5B expression was higher in differentiated tissues compared to pluripotent cells, we wondered if differentiation itself would be sufficient to upregulate WDR5B. To test this hypothesis, we collected RNA from H9 ESCs during a 6-day directed differentiation protocol that uses dual SMAD inhibition to generate neural ectoderm progenitors [43,62,63,64]. A statistically significant increase in WDR5B mRNA was detected by day 2, while levels of WDR5 mRNA decreased (Figure 3A). After 6 days, WDR5B was upregulated approximately 2-fold while WDR5 was downregulated approximately 0.5-fold compared to H9 ESCs. As expected, neural differentiation robustly downregulated the pluripotency marker NANOG and upregulated the neural marker PAX6 (Figure 3A). These results support previous work indicating that WDR5 levels decrease upon differentiation of mouse ESCs [5,61]. To exclude cell line-specific effects, we replicated the experiment using an additional embryonic stem cell line (UCSF4) as well as two induced pluripotent stem cell (iPSC) lines. A similar magnitude of WDR5B upregulation and WDR5 downregulation was observed in all cases (Figure 3B–D). Finally, we wondered if the increase in WDR5B mRNA during neural differentiation would correspond to an increase in WDR5B protein levels. In agreement with our RT-qPCR results (Figure 1E and Figure 3A–D), undifferentiated H9 ESCs produced relatively low levels of WDR5B protein, while neural differentiation increased WDR5B protein levels approximately 5-fold (Figure 3E). These data collectively indicate that WDR5 and WDR5B exhibit directionally opposite changes in expression upon neural differentiation.

### 3.3. Depletion of WDR5B Impairs RPE Cell Proliferation

Since WDR5B is upregulated during ectoderm differentiation, we sought to determine if depletion of WDR5B might impact the physiology of ectoderm-derived cell types. We chose RPE for this study given the existence of robust RPE differentiation protocols that are initiated by dual SMAD inhibition [43,44,64,65] as well as the availability of donor-derived cell lines such as ARPE-19 [66]. WDR5B knockout H9 ESC lines were generated using CRISPR-Cas9 (Figure 2 and Figure S3), and RPE differentiation of two clones per genotype was conducted as previously described [43,44]. During RPE differentiation and after high-density passage to initiate epithelial monolayer formation, no substantial differences between wild-type and WDR5B-knockout lines were apparent. All lines achieved cobblestone morphology and pigmentation typical of mature hESC-RPE (Figure S4A). Similarly, expression of the RPE marker genes BEST1, TYR, RLBP1, and RPE65 as well as the epithelial-to-mesenchymal transition (EMT) marker S100A4 and the proliferation marker MKI67 did not exhibit statistically significant differences by genotype in mature hESC-RPE cultures (Figure S4B,C). We also confirmed that cultures of WDR5B knockout hESC-RPE did not express any detectable WDR5B mRNA (Figure S4D). These data indicate that while WDR5 is required for the maintenance of pluripotency [5], loss of WDR5B in H9 ESCs appears to be tolerated. WDR5B-null ESCs retain the ability to differentiate toward an RPE fate as evidenced by the acquisition of pigmentation, cobblestone morphology, and expression of RPE-specific marker genes.

**Figure 4:**
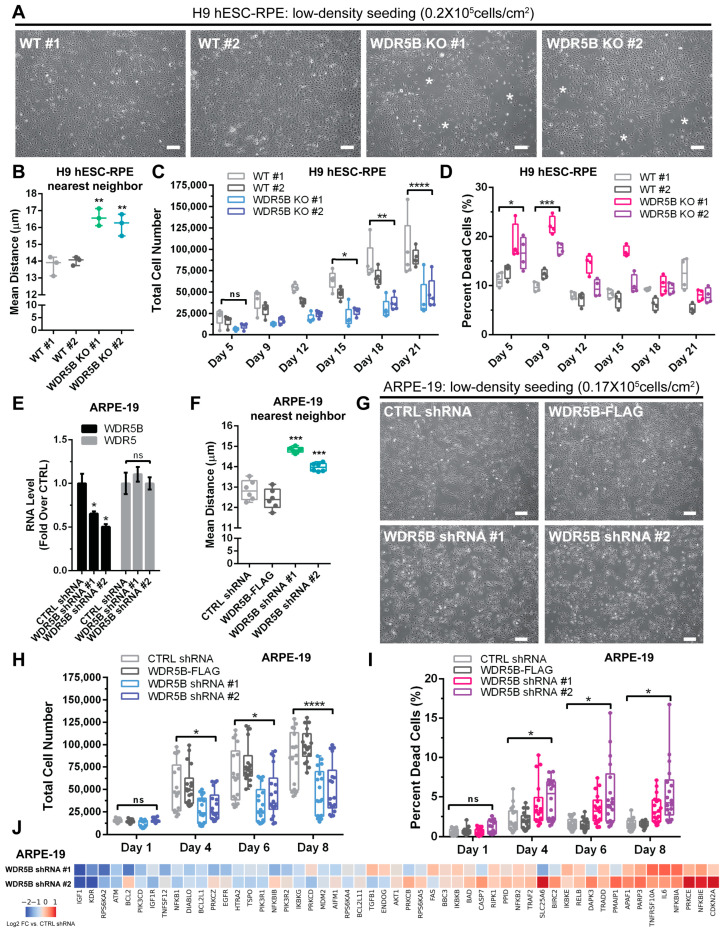
Depletion of WDR5B impairs RPE proliferation. (**A**) Phase contrast images of H9 hESC-RPE at day 9 post-seeding from wild-type (WT #1,2) or WDR5B knockout (WDR5B KO #1,2) clones. White asterisks indicate areas of low cell density. (**B**) Quantification of the sparse growth morphology of WDR5B knockout RPE indicated a larger distance between neighboring cells for both KO clones compared to the wild-type controls (n = 3 fields of view per RPE line, 2391 ± 741 cells per field). (**C**) Loss of WDR5B reduces the number of total cells per well (Hoechst-positive nuclei) compared to wild-type controls (n = 5 biological replicates per group). (**D**) Increased cell death (propidium iodide-positive nuclei) occurred at days 5 to 9 post-seeding in WDR5B knockout RPE compared to wild-type controls. (**E**) Two independent shRNAs reduce WDR5B mRNA levels in ARPE-19 stable lines (*x*-axis) without affecting levels of WDR5. (**F**) Quantification of the sparse growth morphology of WDR5B-depleted ARPE-19 cells indicated a larger distance between neighboring cells for both WDR5B shRNA lines compared to controls or ARPE-19 cells overexpressing WDR5B-FLAG (n = 6 fields of view per RPE line, 2664 ± 577 cells per field). (**G**) Phase contrast images at day 4 post-seeding illustrate the sparse growth morphology of WDR5B-depleted ARPE-19 cell lines compared to controls or ARPE-19 cells overexpressing WDR5B-FLAG. (**H**) Knockdown of WDR5B impairs ARPE-19 proliferation compared to a control shRNA or WDR5B-FLAG overexpression (n = 18 biological replicates per group). (**I**) Increased cell death occurs in WDR5B-depleted ARPE-19 lines between days 4 and 8 post-seeding. (**J**) Heatmap of apoptosis-related gene expression determined by TaqMan RT-qPCR array in two WDR5B knockdown ARPE-19 stable lines at day 8 post-seeding. Values are presented as log2 fold changes relative to the control shRNA. For proliferation and viability, significance was assessed by two-way ANOVA with Tukey’s post hoc, and asterisks indicate the maximum *p*-value where both WDR5B-depleted lines exhibit a significant difference compared to both controls. Error bars, ±SD. Scale bars: 100 µm. ns (not significant), * *p* < 0.05, ** *p* < 0.01, *** *p* < 0.001, **** *p* < 0.0001.

Upregulation of WDR5 is observed in several cancer types, and loss of WDR5 or inhibition of WDR5 interactions at the WIN binding site are sufficient to cause tumor cell apoptosis [21,67,68,69,70,71,72,73]. Accordingly, we wondered if loss of WDR5B might affect RPE cell proliferation. In mammals, RPE cells terminally differentiate during embryogenesis and typically remain quiescent except under pathological scenarios such as PVR [39,40]. RPE proliferation can be initiated in vitro by dissociating and re-seeding RPE at subconfluent density, although recovery of epithelial characteristics diminishes with increasing passage number and is ultimately limited by EMT [42,74,75,76,77,78,79]. To investigate whether loss of WDR5B affects hESC-RPE proliferation, we seeded wild-type and WDR5B-knockout hESC-RPE at low density (20,000 cells/cm^2^) and quantified both total (Hoechst) and non-viable (propidium iodide) nuclei using whole-well imaging. By day 9 post-seeding, wild-type hESC-RPE lines reached confluence while WDR5B-knockout lines exhibited numerous areas of low cell coverage (Figure 4A, asterisks) and a sparse growth morphology (Figure 4B). A statistically significant difference in total nuclei per well emerged in WDR5B-knockout hESC-RPE at day 15 post-seeding in accordance with an elevated number of dead cells observed at earlier timepoints (Figure 4C,D). Cryopreserved banks of all four hESC-RPE lines exhibited ~95% viability immediately post-thaw with no significant differences by genotype, indicating that the increased cell death in WDR5B-knockout hESC-RPE occurred primarily during the first 9 days of culture rather than during cryopreservation or at the time of thaw (Figure S4E).

To assess the effect of WDR5B depletion on RPE proliferation using an independent cell line, we chose spontaneously immortalized ARPE-19 RPE derived from a male human donor [66]. ARPE-19 cells can form epithelial monolayers that exhibit several characteristics of mature RPE yet also retain a limited degree of proliferative capacity [66,77]. However, since ARPE-19 cells seeded at single-cell density are reported to frequently undergo senescence [66], we did not pursue a CRISPR-Cas9 knockout strategy. Instead, we generated ARPE-19 stable lines expressing a control shRNA, two shRNAs targeting WDR5B, or overexpressing WDR5B-FLAG for use in proliferation and viability assays. Both shRNAs reduced the level of WDR5B mRNA by 35–50% without affecting expression of WDR5 (Figure 4E). WDR5B-knockdown ARPE-19 cells also displayed a sparse pattern of cell growth compared to control or WDR5B-FLAG-expressing lines (Figure 4F,G). Accordingly, knockdown of WDR5B impaired proliferation and increased the fraction of non-viable cells over time (Figure 4H,I). In contrast, overexpression of WDR5B-FLAG had little effect on ARPE-19 proliferation or viability (Figure 4H,I).

To better understand the mechanism by which depletion of WDR5B impairs RPE proliferation, we examined apoptosis-related gene expression in WDR5B-knockdown ARPE-19 lines on day 8 post-seeding using an RT-qPCR array. Compared to the control shRNA line, WDR5B-knockdown lines exhibited substantial downregulation of genes related to growth factor signaling such as IGF1, KDR (VEGFR2), IGF1R, and RPS6KA2 as well as slight upregulation of proapoptotic genes including CDKN2A and TNFRSF10A (Figure 4J). These results suggest that reduced growth factor signaling may contribute to the impaired RPE proliferation phenotype caused by WDR5B depletion.

### 3.4. Genomic WDR5 Binding Sites Exhibit Minimal WDR5B Enrichment

**Figure 5:**
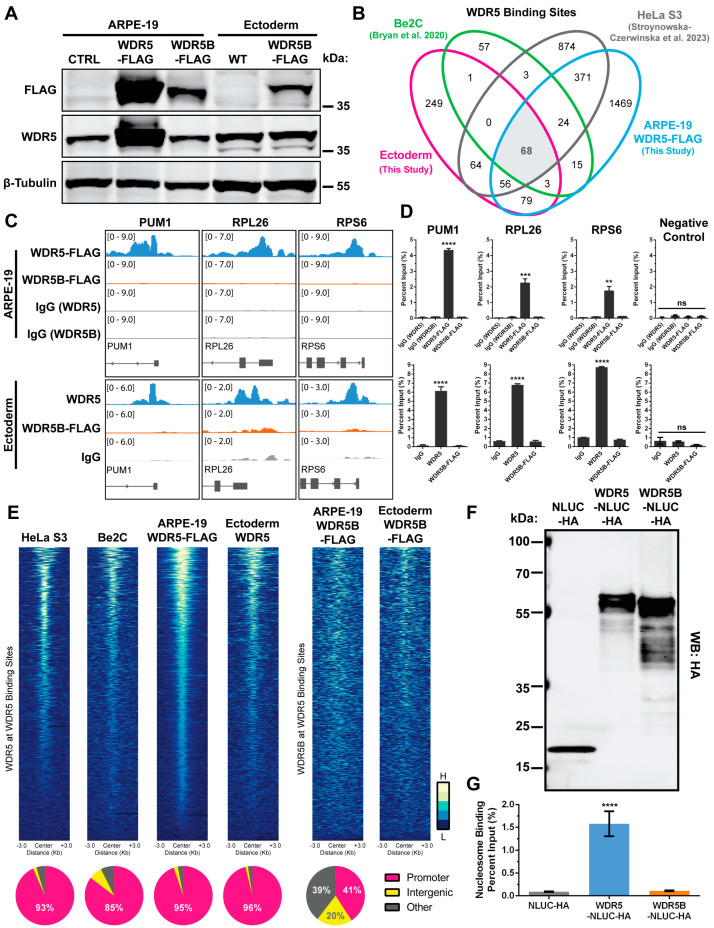
WDR5B enrichment was not detected at WDR5 genomic binding sites. (**A**) Expression of WDR5, WDR5-FLAG, and WDR5B-FLAG were verified in CUT&RUN samples by Western blot. (**B**) Venn diagram indicates overlap between WDR5 peaks (*p* < 0.001) in this study and previous datasets. No overlapping WDR5B peaks were detected across cell lines [72,80]. (**C**) (Top) Genomic views of representative WDR5-bound genes PUM1, RPL26, and RPS6 in ARPE-19 cells. WDR5-FLAG binding is abundant near the TSS with minimal WDR5B-FLAG or IgG signal present. (Bottom) Similar results were observed for endogenous WDR5 and WDR5B in ectoderm. Genomic views depict 3 kb regions. (**D**) (Top) CUT&RUN-qPCR performed on ARPE-19 samples confirmed binding of WDR5-FLAG near the TSS of PUM1, RPL26, and RPS6, but not at an intergenic negative control region. WDR5B-FLAG did not bind to any of these genomic sites. (Bottom) Similar CUT&RUN-qPCR results were observed for ectoderm. (**E**) Heatmaps of WDR5 and WDR5B signal intensity around WDR5 peaks (1110 peaks, q < 0.05). Signal intensity was normalized to IgG as well as for read coverage (bins per million mapped reads). Heatmaps represent 300 bp bins ± 3 kb from peak centers. (Bottom) At least 85% of WDR5 peaks (*p* < 0.001) in all cell lines were within gene promoters while most WDR5B peaks were not. (**F**) In vitro translation of NLUC-HA, WDR5-NLUC-HA, and WDR5B-NLUC-HA produces proteins of the expected sizes as verified by Western blot. (**G**) WDR5 binding and recovery of unmodified recombinant nucleosomes in vitro is significantly greater than that of WDR5B or NLUC-HA alone. For CUT&RUN-qPCR and in vitro nucleosome binding, n = 3 experimental replicates per group, and significance was assessed by one-way ANOVA with Tukey’s post hoc. Error bars, ±SD. ns (not significant), ** *p* < 0.01, *** *p* < 0.001, **** *p* < 0.0001.

Since the chromatin binding profile of WDR5 has been characterized in several human and mouse cell lines [5,72,80], we wondered if WDR5B might also associate with chromatin. To test this hypothesis we performed genome-wide chromatin profiling for WDR5 and WDR5B in two cell lines, ARPE-19 and H9-derived neural ectoderm, using the Cleavage Under Targets and Release Using Nuclease (CUT&RUN) technique [49,81]. Previously published WDR5 CUT&RUN data from HeLa S3 cells [80] and WDR5 ChIP-seq data from human Be2C neuroblastoma cells [72] were also obtained for comparison and analyzed using identical workflows. Protein expression of WDR5 and WDR5B was verified by Western blot, although equal multiplicity of infection (MOI) transduction of ARPE-19 cells resulted in ~5-fold higher WDR5-FLAG levels compared to WDR5B-FLAG (Figure 5A). CUT&RUN for endogenous WDR5 and overexpressed WDR5-FLAG yielded hundreds to thousands of peaks at a threshold of *p* < 0.001 (Figure 5B). In contrast, no significant WDR5B-FLAG peaks common to both cell lines were detected using the same FLAG antibody, and no WDR5B peaks in either cell line reached significance at a false discovery rate of q < 0.05. Overlap analysis of WDR5 peaks (*p* < 0.001) revealed 68 sites common to all four datasets (Figure 5B), which represent 73 of the 94 previously identified WDR5-bound consensus genes when overlapping promoters are considered [72]. This overlap was observed despite differences in cell line, chromatin profiling method, and WDR5-targeting antibody, indicating good reproducibility.

To verify these findings, we performed CUT&RUN-qPCR near the TSS of three representative WDR5-bound genes, PUM1, RPL26, and RPS6, in ARPE-19 and ectoderm cells. Our CUT&RUN data indicate clear WDR5 peaks in these regions with minimal WDR5B or control IgG signal (Figure 5C). CUT&RUN-qPCR confirmed the specific enrichment of WDR5 but not WDR5B at these locations (Figure 5D). Since no highly significant WDR5B peaks were detected (q < 0.05), we plotted heatmaps to visualize WDR5 and WDR5B signal at the 1110 WDR5-bound sites observed in at least one dataset (Figure 5E, top). While signal from WDR5 ChIP-seq and CUT&RUN was enriched at WDR5 peaks, minimal WDR5B CUT&RUN signal above background was observed at these sites (Figure 5E, top). Consistent with the established binding profile of WDR5 near the TSS of target genes [72], ≥85% of WDR5 peaks (*p* < 0.001) in all cell lines were within promoters (Figure 5E, bottom). However, 59% of the 76 combined low-confidence WDR5B peaks from ARPE-19 and ectoderm were outside of promoter regions (Figure 5E, bottom). Given the apparent lack of WDR5B-bound loci detected by CUT&RUN, we wondered if WDR5B would exhibit any interaction with histones or nucleosomes in vitro, as has been demonstrated for WDR5 [82,83]. To address this question, we used in vitro transcription and translation to produce nanoluciferase-HA (NLUC-HA) tagged WDR5 and WDR5B proteins followed by HA tag affinity purification with magnetic beads (Figure 5F). Incubation of these bead-bound proteins with unmodified recombinant nucleosomes in CUT&RUN buffer revealed significant nucleosome recovery by WDR5-NLUC-HA but not by WDR5B-NLUC-HA or NLUC-HA alone (Figure 5G). Together, these data support the conclusion that WDR5B exhibits substantially different chromatin binding characteristics compared to its close homolog WDR5.

## 4. Discussion

According to some estimates, more than 30% of human proteins remain largely unexplored beyond the knowledge of a primary amino acid sequence [84,85,86]. This study examines one such protein, WDR5B, which exhibits 86% sequence identity to the well-studied chromatin-associated protein WDR5. We find that the gene encoding WDR5B appears to be present only in placental mammals and is upregulated upon neural differentiation. In contrast, WDR5 is highly conserved from yeast to humans and is more abundantly expressed in pluripotent cell types compared to differentiated tissues from ectoderm, mesoderm, or endoderm (Figures S1 and S2). Consistent with elevated expression of WDR5B in differentiated cells, loss or depletion of WDR5B impaired proliferation and increased cell death in multiple lines of cultured RPE, a differentiated cell type of neuroectodermal origin. Since WDR5 and WDR5B both localize to the nucleus, we performed genome-wide as well as targeted chromatin binding analyses for each protein. The WDR5 chromatin binding sites observed in this study substantially recapitulate prior work [72,80]. However, WDR5B did not appear to robustly associate with chromatin under the same experimental conditions. In vitro binding of WDR5, but not WDR5B, to recombinant nucleosomes also suggests that WDR5B may exhibit little intrinsic chromatin binding ability.

Given the high degree of sequence homology between WDR5 and WDR5B, further comparative studies could provide important insights regarding both proteins. WDR5 associates with histone H3, KIF2A, and the MLL/SET catalytic subunits via its central arginine-binding WIN cavity [83,87,88], while its association with RBBP5 and MYC occur via the hydrophobic WBM pocket [21,89]. The development of small molecule inhibitors of interactions at both the WIN and WBM sites also revealed additional WDR5 binding proteins that specifically associate with each site [90,91]. A divergence in the chromatin binding properties of WDR5 and WDR5B is especially intriguing since critical residues at both the WIN and WBM binding pockets of WDR5 are conserved in WDR5B (Figure 1D). It is also unclear whether small molecule inhibitors of WDR5 similarly bind to WDR5B. We cannot exclude the possibility that WDR5B associates weakly or transiently with chromatin or that modified CUT&RUN conditions are required to detect WDR5B-chromatin binding. WDR5B might require specific chromatin modifications, RNA, or other chromatin-associated factors for stable chromatin binding, which could similarly explain its apparent inability to bind recombinant nucleosomes in vitro (Figure 5G). Furthermore, WDR5B may associate with highly repetitive or anomalous genomic regions that are typically excluded from CUT&RUN and ChIPseq analyses for technical reasons [51,92]. However, our results indicate that WDR5B does not share the same chromatin binding characteristics as WDR5, and that WDR5B does not robustly associate with WDR5-bound loci under these experimental conditions.

Alternatively, the lack of prominent WDR5B-bound loci might indicate a chromatin-independent function. Nearly ~1% of the human proteome is comprised of WD40 repeat-containing proteins [93]. Despite sharing a well-conserved β-propeller structure they interact with a diverse array of target molecules including phosphoserine and phosphothreonine residues [94,95], ubiquitin [96], DNA photolesions [97], lncRNAs [28], and the phosphoinositide lipids PI(3)P and PI(3,5)P2 [98]. Many surfaces of the β-propeller structure have been shown to facilitate interactions including the top, bottom, sides, and central cavity [93,99]. In the absence of known WDR5B interacting partners, the exact biochemical processes facilitated by WDR5B remain difficult to determine. However, given the lack of in vitro binding between WDR5B and nucleosomes (Figure 5G), which include a WIN motif in the histone H3 N-terminal tail, it appears less likely that WDR5B might form complexes with other proteins that require an intact WIN-binding site such as KIF2A or the MLL/SET catalytic subunits. While the large number of potential WDR5B interacting partners presents a challenge, successfully identifying them could provide valuable insight into the precise cellular function of WDR5B.

Upregulation of WDR5B upon neural differentiation (Figure 3) as well as the elevated expression of WDR5B in differentiated tissues compared to pluripotent cells also stands in contrast to WDR5, which is highly expressed in stem cells and required for pluripotency [5,61]. Consistent with this pattern of expression, complete loss of WDR5B was tolerated in H9 ESCs and had no apparent effect on the ability of these cells to differentiate into hESC-RPE (Figure S4). However, WDR5B-null hESC-RPE as well as WDR5B-depleted ARPE-19 RPE exhibited impaired growth in response to low-density seeding, which elicits a well characterized EMT response in RPE [42,76,78]. Although WDR5B depletion reduced the viability of both hESC-RPE and ARPE-19 cells, the onset and duration of this effect varied (Figure 4D,I), perhaps due to known differences in the proliferative ability, stress response, or transcriptome of ARPE-19 cells compared to hESC-RPE [100,101]. Profiling of apoptosis-related gene expression in WDR5B-depleted ARPE-19 lines revealed reduced expression of genes involved in growth factor signaling including IGF1, KDR (VEGFR2), RPS6KA2, and IGF1R (Figure 4J), which typically promote the survival and proliferation of RPE as well as other cell types [102,103,104,105,106]. While WDR5B depletion may elicit these changes either directly or via indirect effects on upstream pathways, they are consistent with the observed phenotype of impaired proliferation and reduced viability. Interestingly, a genome-wide CRISPR-Cas9 screen found that loss of WDR5B reduced the cellular fitness of several tumor cell lines [107]. Both the cell type specificity and precise mechanism by which WDR5B affects proliferation and viability remain unclear and warrant further investigation.

Since WDR5B depletion impairs the viability and proliferation of RPE undergoing EMT (Figure 4), the relevance of WDR5B to the EMT process may also merit consideration. Data from a single cell RNA sequencing study of in vitro RPE differentiation indicate that WDR5B expression is highest on day 7 of RPE differentiation, and that WDR5B is also enriched in clusters associated with mesenchyme and cranial neural crest cells [108]. These data are consistent with our finding that neural progenitors upregulate WDR5B (Figure 3) and raise the question of whether WDR5B might play a role in developmental EMT, which is known to occur during gastrulation and neural crest delamination [109,110,111,112].

## 5. Conclusions

In summary, this initial examination of WDR5B reveals substantial differences in evolutionary conservation, expression pattern, and chromatin binding properties compared to WDR5, its closest homolog. Additional work will be required to identify the relevant interacting partners of WDR5B as well as the cellular processes in which it participates. While WDR5 is highly conserved and considered an essential gene in human cells [113,114,115], the presence of WDR5B only in the genomes of placental mammals may indicate involvement in a function specific to this clade of organisms. Finally, a deeper understanding of how WDR5B depletion slows RPE proliferation during EMT could lead to new targets for PVR, which often requires surgical intervention [39,116].

## Data Availability

CUT&RUN sequencing data from this study have been deposited in the NCBI Gene Expression Omnibus (GEO) repository with the accession number GSE244882.

## Supplementary Materials

The following supporting information can be downloaded at https://www.mdpi.com/article/10.3390/cells13141189/s1, Figure S1: WDR5B is conserved among eutherian mammals; Figure S2: Expression levels of WDR5 and WDR5B are inversely correlated in NCBI Gene Expression Omnibus (GEO) transcriptome datasets curated by StemMapper; Figure S3: Verification of CRISPR-Cas9 genome editing; Figure S4: WDR5B knockout hESC-RPE exhibit normal RPE morphology and expression of RPE marker genes when seeded at high density; Table S1: Oligonucleotide sequences.
