# Supplementary material for: Initial Characterization of WDR5B Reveals a Role in the Proliferation of Retinal Pigment Epithelial Cells
Source: Cells. 2024 Jul 13;13(14):1189. doi: 10.3390/cells13141189 (PMC11275010; doi:10.3390/cells13141189)
Supplement: Supplementary file 1 [file cells-13-01189-s001.zip › cells-3028012-supplementary.pdf]

# Figure S1

## A

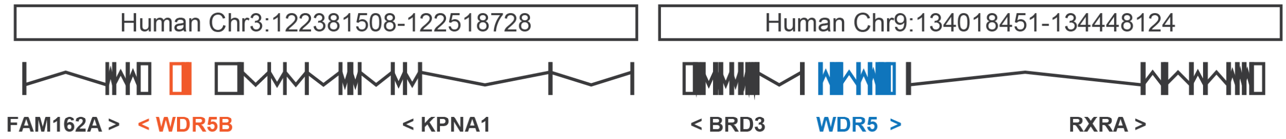


## B

| | | Species | WDR5B locus | | | WDR5 locus | | | Location and Genome Version | |
|---|---|---|---|---|---|---|---|---|---|---|
| | | | Upstream | | Downstream | Upstream | | Downstream | WDR5B locus | WDR5 locus |
| | | | FAM162A | WDR5B | KPNA1 | BRD3 | WDR5 | RXRA | | |
| Mammals | Primates | Chimpanzee (Pan troglodytes) | ✓ 99.4% | ✓ 99.7% | ✓ 99.8% | ✓ 99.7% | ✓ 100.0% | ✓ 100.0% | Chromosome 3 (mPanTro3-v1.1) | Chromosome 9 (mPanTro3-v1.1) |
| | | Gorilla (Gorilla gorilla gorilla) | ✓ 99.4% | ✓ 99.1% | ✓ 99.8% | ✓ 99.6% | ✓ 100.0% | ✓ 99.8% | Chromosome 3 (mGorGor1-v1.1-0.2) | Chromosome 9 (mGorGor1-v1.1-0.2) |
| | | Orangutan (Pongo abelii) | ✓ 94.8% | ✓ 99.1% | ✓ 99.8% | ✓ 99.6% | ✓ 100.0% | ✓ 99.8% | Chromosome 3 (mPonAbe1-v1.1) | Chromosome 9 (mPonAbe1-v1.1) |
| | | Gibbon (Nomascus leucogenys) | ✓ 97.4% | ✓ 98.8% | ✓ 99.8% | ✓ 99.3% | ✓ 100.0% | ✓ 100.0% | Chromosome 21 (Asia_NLE_v1) | Chromosome 8 (Asia_NLE_v1) |
| | | Rhesus Macaque (Macaca mulatta) | ✓ 88.3% | ✓ 97.3% | ✓ 99.8% | ✓ 99.0% | ✓ 100.0% | ✓ 100.0% | Chromosome 2 (Mmul_10) | Chromosome 15 (Mmul_10) |
| | Artiodactyla, Perissodactyla, and Carnivora | Pig (Sus scrofa) | ✓ 83.9% | ✓ 91.8% | ✓ 99.4% | ✓ 92.6% | ✓ 99.1% | ✓ 97.5% | Chromosome 13 (Sscrofa11.1) | Chromosome 1 (Sscrofa11.1) |
| | | Giant panda (Ailuropoda melanoleuca) | ✓ 81.0% | ✓ 91.8% | ✓ 72.7% | ✓ 94.3% | ✓ 100.0% | ✓ 98.2% | Chromosome 1 (ASM200744v2) | Chromosome 7 (ASM200744v2) |
| | | Sheep (Ovis aries) | ✓ 82.6% | ✓ 91.5% | ✓ 99.4% | ✓ 92.6% | ✓ 99.7% | ✓ 97.3% | Chromosome 1 (ARS-UI_Ramb_v2.0) | Chromosome 3 (ARS-UI_Ramb_v2.0) |
| | | Dolphin (Tursiops truncatus) | ✓ 83.9% | ✓ 91.2% | ✓ 99.1% | ✓ 93.5% | ✓ 99.7% | ✓ 97.2% | Chromosome 4 (mTurTru1.mat.Y) | Chromosome 6 (mTurTru1.mat.Y) |
| | | Horse (Equus caballus) | ✓ 88.4% | ✓ 91.2% | ✓ 99.1% | ✓ 94.2% | ✓ 100.0% | ✓ 99.2% | Chromosome 19 (EquCab3.0) | Chromosome 25 (EquCab3.0) |
| | | Donkey (Equus asinus) | ✓ 87.1% | ✓ 91.2% | ✓ 99.1% | ✓ 94.3% | ✓ 99.7% | ✓ 99.5% | Chromosome 5 (ASM1607732v2) | Chromosome 10 (ASM1607732v2) |
| | | Cow (Bos taurus) | ✓ 84.0% | ✓ 91.2% | ✓ 99.3% | ✓ 92.0% | ✓ 100.0% | ✓ 97.5% | Chromosome 1 (ARS-UCD1.2) | Chromosome 11 (ARS-UCD1.2) |
| | | Hippo (Hippopotamus amphibius kiboko) | ✓ 81.3% | ✓ 91.2% | ✓ 99.4% | ✓ 94.4% | ✓ 100.0% | ✓ 96.4% | Chromosome 10 (mHipAmp2.hap2) | Chromosome 2 (mHipAmp2.hap2) |
| | | Dog (Canis lupus familiaris) | ✓ 80.6% | ✓ 91.2% | ✓ 99.4% | ✓ 94.4% | ✓ 100.0% | ✓ 97.9% | Chromosome 33 (ROS_Cfam_1.0) | Chromosome 9 (ROS_Cfam_1.0) |
| | | Cat (Felis catus) | ✓ 84.0% | ✓ 90.6% | ✓ 99.4% | ✓ 92.8% | ✓ 100.0% | ✓ 99.2% | Chromosome C2 (F.catus_Fca126_mat1.0) | Chromosome D4 (F.catus_Fca126_mat1.0) |
| | Rodents and lagomorphs | Hamster (Mesocricetus auratus) | ✓ 81.3% | ✓ 86.3% | ✓ 98.5% | ✓ 95.6% | ✓ 100.0% | ✓ 97.9% | NW_024429192 Unplaced Scaffold (BCM_Maur_2.0) | NW_024429189 Unplaced Scaffold (BCM_Maur_2.0) |
| | | Guinea Pig (Cavia porcellus) | ✓ 74.2% | ✓ 85.2% | ✓ 99.1% | ✓ 95.2% | ✓ 100.0% | ✓ 98.6% | NT_176332 Unplaced Scaffold (Cavpor3.0) | NT_176401 Unplaced Scaffold (Cavpor3.0) |
| | | Rat (Rattus norvegicus) | ✓ 80.6% | ✓ 84.5% | ✓ 98.8% | ✓ 95.2% | ✓ 100.0% | ✓ 98.7% | Chromosome 11 (mRatBN7.2) | Chromosome 3 (mRatBN7.2) |
| | | Mouse (Mus musculus) | ✓ 78.7% | ✓ 83.3% | ✓ 97.8% | ✓ 94.4% | ✓ 100.0% | ✓ 97.4% | Chromosome 16 (GRCm39 C57BL/6J) | Chromosome 2 (GRCm39 C57BL/6J) |
| | | American Pika (Ochotona princeps) | ✓ 78.7% | ✓ 81.9% | ✓ 99.4% | ✓ 92.1% | ✓ 98.2% | ✓ 94.4% | Chromosome 3 (mOchPri1.hap1) | Chromosome 14 (mOchPri1.hap1) |
| | Monotremes and marsupials | Echidna (Tachyglossus aculeatus) | ✓ 70.8% | --- | ✓ 92.0% | ✓ 91.3% | ✓ 97.9% | ✓ 96.8% | Chromosome 16 (mTacAcu1.pri) | Chromosome 4 (mTacAcu1.pri) |
| | | Platypus (Ornithorhynchus anatinus) | ✓ 75.3% | --- | ✓ 92.4% | ✓ 91.5% | ✓ 97.6% | ✓ 98.3% | Chromosome 16 (mOrnAna1.pri.v4) | Chromosome 4 (mOrnAna1.pri.v4) |
| | | Possum (Monodelphis domestica) | ✓ 64.2% | --- | ✓ 97.0% | ✓ 91.3% | ✓ 97.3% | ✓ 97.8% | Chromosome 4 (mMonDom1.pri) | Chromosome 1 (mMonDom1.pri) |
| | | Tasmanian devil (Sarcophilus harrisii) | ✓ 65.2% | --- | ✓ 97.3% | ✓ 91.3% | ✓ 97.3% | ✓ 96.4% | Chromosome 3 (mSarHar1.11) | Chromosome 2 (mSarHar1.11) |
| Birds, reptiles, and amphibians | | Common toad (Bufo bufo) | ✓ 53.3% | --- | ✓ 92.0% | ✓ 73.2% | ✓ 97.3% | ✓ 85.9% | Chromosome 3 (aBufBuf1.1) | Chromosome 8 (aBufBuf1.1) |
| | | Komodo dragon (Varanus komodoensis) | ✓ 48.3% | --- | ✓ 95.2% | ✓ 85.7% | ✓ 97.3% | ✓ 92.1% | NW_025336223 Unplaced Scaffold (ASM479886v1) | NW_025336442 Unplaced Scaffold (ASM479886v1) |
| | | Chicken (Gallus gallus) | ✓ 54.7% | --- | ✓ 95.7% | ✓ 91.2% | ✓ 97.0% | ✓ 96.4% | Chromosome 1 (bGalGal1.mat.broiler.GRCg7b) | Chromosome 17 (bGalGal1.mat.broiler.GRCg7b) |
| | | Turkey (Meleagris gallopavo) | ✓ 53.6% | --- | ✓ 96.1% | ✓ 90.7% | ✓ 97.0% | ✓ 96.4% | Chromosome 1 (Turkey_5.1) | Chromosome 19 (Turkey_5.1) |
| | | Peregrine falcon (Falco peregrinus) | ✓ 55.0% | --- | ✓ 96.3% | ✓ 91.2% | ✓ 97.0% | ✓ 95.1% | Chromosome 6 (bFalPer1.pri) | Chromosome 1 (bFalPer1.pri) |
| | | Ostrich (Struthio camelus australis) | ✓ 56.9% | --- | ✓ 96.3% | ✓ 91.4% | ✓ 97.0% | ✓ 96.4% | NW_009271951.1 Unplaced Scaffold (ASM69896v1) | NW_009270502.1 Unplaced Scaffold (ASM69896v1) |
| | | Zebra finch (Taeniopygia guttata) | ✓ 49.3% | --- | ✓ 95.7% | ✓ 92.2% | ✓ 97.0% | ✓ 94.6% | Chromosome 1 (bTaeGut1.4.pri) | Chromosome 17 (bTaeGut1.4.pri) |
| | | Clawed frog (Xenopus tropicalis) | ✓ 60.2% | --- | ✓ 94.1% | ✓ 74.6% | ✓ 97.0% | ✓ 85.2% | Chromosome 2 (UCB_Xtro_10.0) | Chromosome 8 (UCB_Xtro_10.0) |
| | | Painted turtle (Chrysemys picta bellii) | ✓ 59.6% | --- | ✓ 95.9% | ✓ 90.2% | ✓ 96.7% | ✓ 95.9% | NW_024885710 Unplaced Scaffold (Chrysemys_picta_BioNano-3.0.4) | Chromosome 20 (Chrysemys_picta_BioNano-3.0.4) |
| | | Garter snake (Thamnophis elegans) | ✓ 29.8% | --- | ✓ 94.4% | ✓ 86.4% | ✓ 96.7% | ✓ 91.7% | Chromosome 3 (rThaEle1.pri) | Chromosome 16 (rThaEle1.pri) |
| Fish | | Spotted gar (Lepisosteus oculatus) | ✓ 45.3% | --- | ✓ 86.0% | ✓ 84.8% | ✓ 96.1% | ✓ 89.3% | Chromosome LG3 (LepOcu1) | Chromosome LG21 (LepOcu1) |
| | | Piranha (Pygocentrus nattereri) | ✓ 51.5% | --- | ✓ 84.7% | ✓ 74.4% | ✓ 95.8% | ✓ 89.2% | Chromosome 24 (fPygNat1.pri) | Chromosome 16 (fPygNat1.pri) |
| | | Zebrafish (Danio rerio) | ✓ 49.5% | --- | ✓ 84.6% | ✓ 75.0% | ✓ 95.8% | ✓ 89.1% | Chromosome 24 (GRCz11) | Chromosome 21 (GRCz11) |
| | | Atlantic herring (Clupea harengus) | ✓ 49.1% | --- | ✓ 82.5% | ✓ 72.6% | ✓ 95.5% | ✓ 82.6% | Chromosome 17 (Ch_v2.0.2) | Chromosome 12 (Ch_v2.0.2) |
| | | Great white shark (Carcharodon carcharias) | ✓ 51.6% | --- | ✓ 87.4% | ✓ 72.5% | ✓ 94.6% | ✓ 89.7% | Chromosome 18 (sCarCar2.pri) | Chromosome 8 (sCarCar2.pri) |
| | | Pufferfish (Takifugu rubripes) | ✓ 47.0% | --- | ✓ 83.4% | ✓ 71.0% | ✓ 94.3% | ✓ 84.3% | Chromosome 10 (fTakRub1.2) | Chromosome 6 (fTakRub1.2) |

**Figure S1.** WDR5B is conserved among eutherian mammals. (**A**) Schematic illustrations of the human WDR5B and WDR5 genomic loci including the nearest annotated upstream and downstream protein coding genes. Arrows indicate the direction of transcription, and vertical bars indicate exons. Distances between genes are not to scale. (**B**) A table of 40 vertebrates indicates the maximum protein sequence identity (%) compared to the human Refseq homolog for each protein as determined by NCBI blastp searches against databases of all annotated proteins for each organism. As expected, collinearity of the WDR5 locus is well conserved in all vertebrate species examined. While collinearity of the genes upstream and downstream of human WDR5B (FAM162A and KPNA1) is also conserved among all vertebrate genomes examined, a gene corresponding to WDR5B is only annotated in the 20 eutherian mammals. Check marks and dashes denote the presence or absence, respectively, of a blastp hit in the expected collinear order for each organism and protein. Chromosome or genomic scaffold locations as well as genome versions for each locus are indicated in the rightmost columns.

## Figure S2

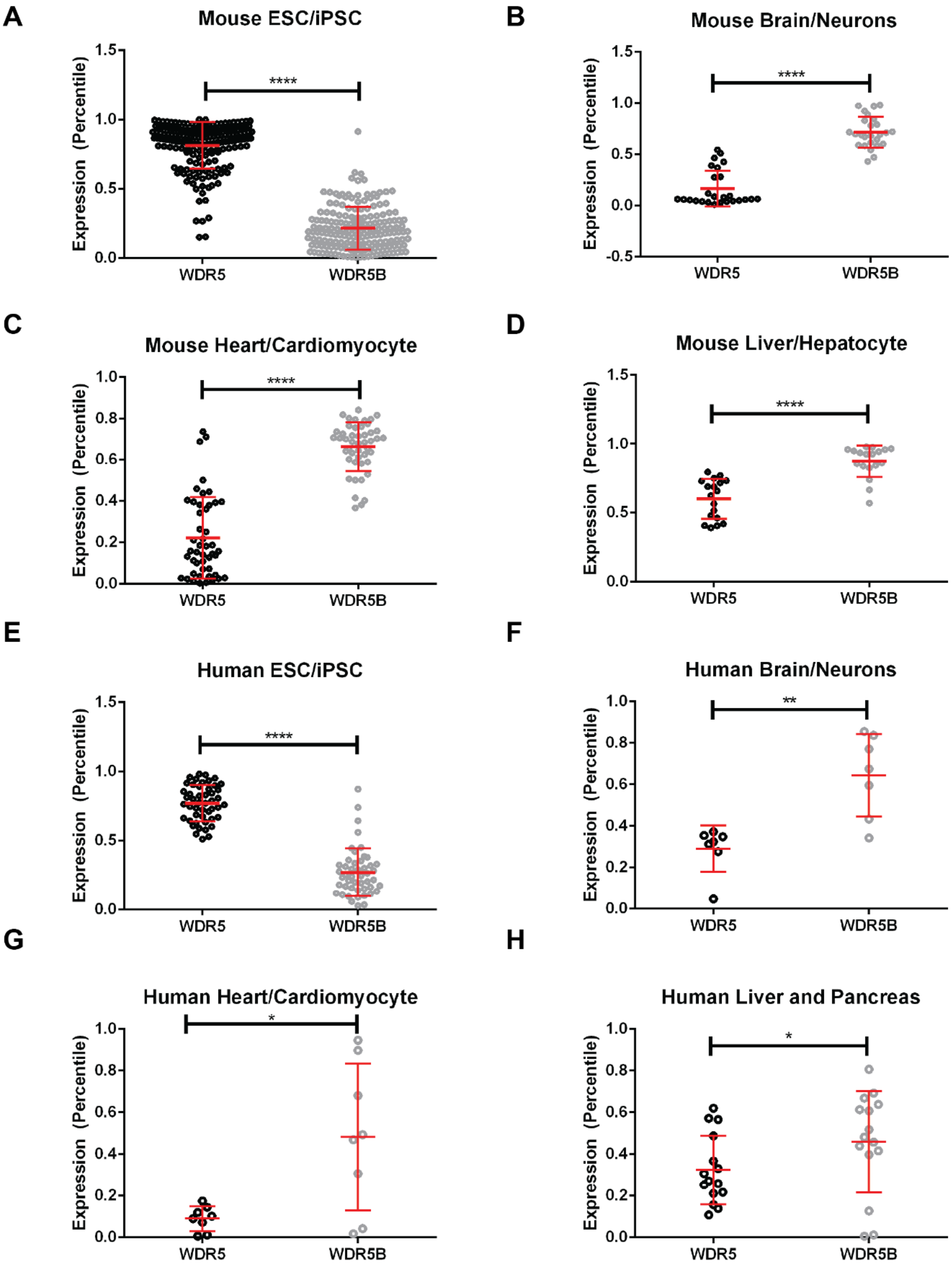
A
Mouse ESC/iPSC
B
Mouse Brain/Neurons
C
Mouse Heart/Cardiomyocyte
D
Mouse Liver/Hepatocyte
E
Human ESC/iPSC
F
Human Brain/Neurons
G
Human Heart/Cardiomyocyte
H
Human Liver and Pancreas
Expression (Percentile)
WDR5
WDR5B
****
**
*

**Figure S2.** Expression levels of WDR5 and WDR5B are inversely correlated in NCBI Gene Expression Omnibus (GEO) transcriptome datasets curated by StemMapper. (**A**) Compared to all other tissues, Mouse ESC and iPSC samples ranked in the 81$^{st}$ percentile for WDR5 expression and in the 22$^{nd}$ percentile for WDR5B expression (n=177 mouse ESC and iPSC transcriptomes). (**B**) Mouse brain and other neural samples had a mean rank of 17$^{th}$ percentile for WDR5 expression and 72$^{nd}$ percentile for WDR5B expression (n=26 mouse neural transcriptomes). (**C**) Mouse heart and cardiomyocyte samples had a mean rank of 22$^{nd}$ percentile for WDR5 expression and 66$^{th}$ percentile for WDR5B expression (n=47 mouse heart and cardiomyocyte transcriptomes). (**D**) Mouse liver and hepatocyte samples had a mean rank of 60$^{th}$ percentile for WDR5 expression and 87$^{th}$ percentile for WDR5B expression (n=18 mouse liver and hepatocyte transcriptomes). (**E**) Similar results were observed for human transcriptomes, where human ESC and iPSC samples ranked in the 77$^{th}$ percentile for WDR5 expression and in the 27$^{th}$ percentile for WDR5B expression (n=48 human ESC and iPSC transcriptomes). (**F**) Human brain and other neural samples had a mean rank of 29$^{th}$ percentile for WDR5 expression and 64$^{th}$ percentile for WDR5B expression (n=7 human neural transcriptomes). (**G**) Human heart and cardiomyocyte samples had a mean rank of 9$^{th}$ percentile for WDR5 expression and 48$^{th}$ percentile for WDR5B expression (n=8 human heart and cardiomyocyte transcriptomes). (**D**) Human liver and pancreas samples had a mean rank of 32$^{nd}$ percentile for WDR5 expression and 46$^{th}$ percentile for WDR5B expression (n=15 human liver and pancreas transcriptomes). All statistical analyses were conducted using the Kolmogorov-Smirnov test since some groups had unequal variance. Asterisks indicate significance as follows: **** ($p < 0.0001$), ** ($p<0.01$), * ($p<0.05$). Error bars are ±S.D.

# Figure S3

**A**

H9 ESC WDR5B-FLAG

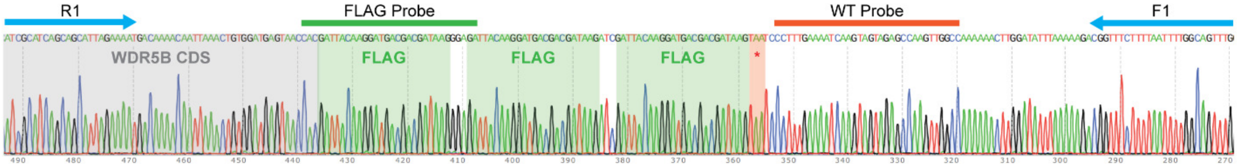


**B**

HT-1080 WDR5B-FLAG

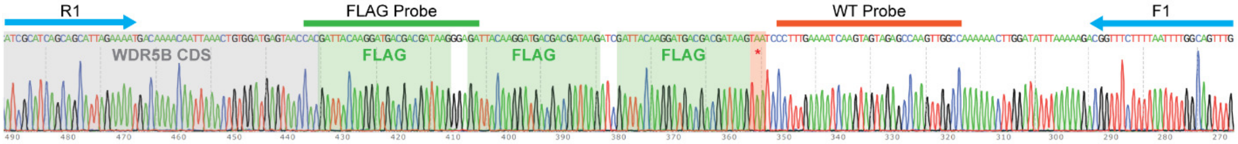


**C**

H9 ESC WDR5B KO #1

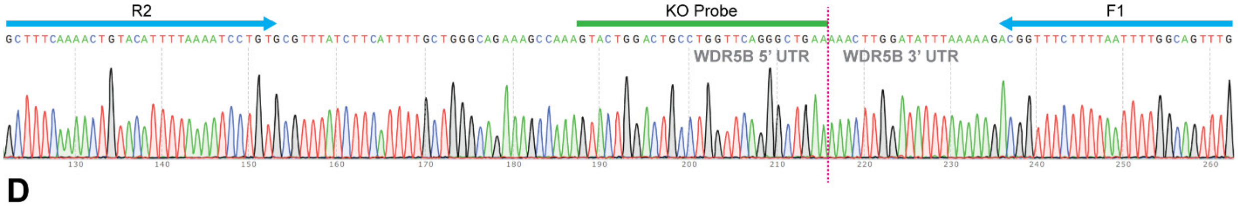


**D**

H9 ESC WDR5B KO #2

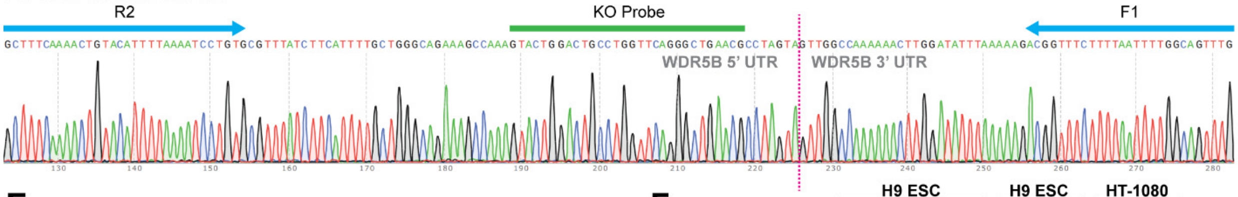


**E**

| | Expected PCR Product Size (bp) | | |
|---|---|---|---|
| **Allele** | **F1+R1** | **F1+R1+R2** | **F2+R3** |
| WT | 146 | 146, 1209* | 1376 |
| KO | - | 160 | 327 |
| FLAG | 224 | 224, 1287* | 1454 |
| ** Not efficiently produced with extension time <1min* | | | |

**F**

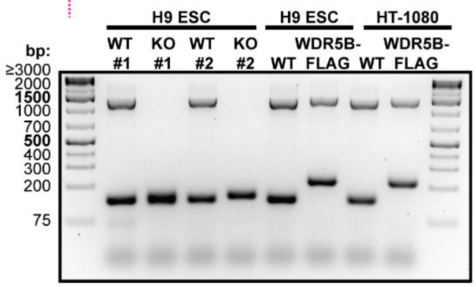


**3% agarose, PCR Primers: F1+R1+R2, 2 min extension time**
**WT**: 146bp + 1209bp **KO**: 160bp **FLAG**: 224bp + 1287bp

**G**

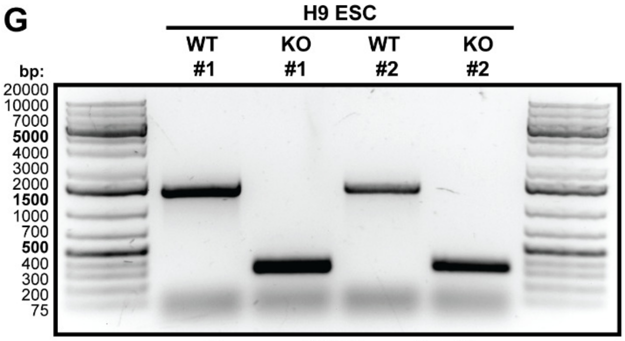


**1% agarose, PCR Primers: F2 + R3**
**WT**: 1376bp **KO**: 327bp

**H**

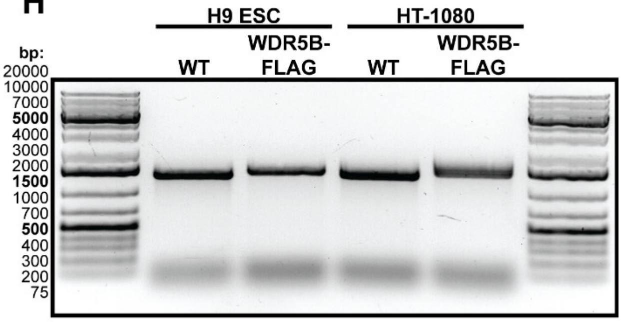


**1% agarose, PCR Primers: F2 + R3**
**WT**: 1376bp **FLAG**: 1454bp

**Figure S3.** Verification of CRISPR-Cas9 genome editing. (**A**) Annotated Sanger sequencing traces for 3XFLAG-tagged WDR5B amplified from the gDNA of the H9 ESC WDR5B-FLAG clonal cell line. (**B**) Annotated Sanger sequencing traces for 3XFLAG-tagged WDR5B amplified from the gDNA of the HT-1080 WDR5B-FLAG clonal cell line. (**C**) Annotated Sanger sequencing traces for the WDR5B-null locus amplified from the gDNA of the H9 ESC WDR5B KO #1 clonal cell line. (**D**) Annotated Sanger sequencing traces for the WDR5B-null locus amplified from the gDNA of the H9 ESC WDR5B KO #2 clonal cell line. For annotated sequencing results, shaded regions and text within the traces indicate sequence features, annotations above the sequence indicate primer or probe binding sites, and dotted vertical lines indicate sites of non-homologous end joining (NHEJ). (**E**) Table of expected PCR product sizes in base pairs (bp) for each combination of alleles and primers. PCR products >1000 bp are not efficiently produced in the presence of shorter amplicons unless PCR extension time exceeds 1 min. (**F**) Primers F1, R1, and R2 produced PCR products of the expected sizes from the gDNA of H9 ESC and HT-1080 wild type and CRISPR-Cas9 edited cell lines. (**G**) Primers F2 and R3, which flank the CRISPR-Cas9 edited region, produced PCR products of the expected sizes from the gDNA of H9 ESC wild type and WDR5B-null clones. (**H**) Primers F2 and R3 also produced PCR products of the expected sizes from the gDNA of H9 ESC and HT-1080 WDR5B-FLAG clones.

## Figure S4

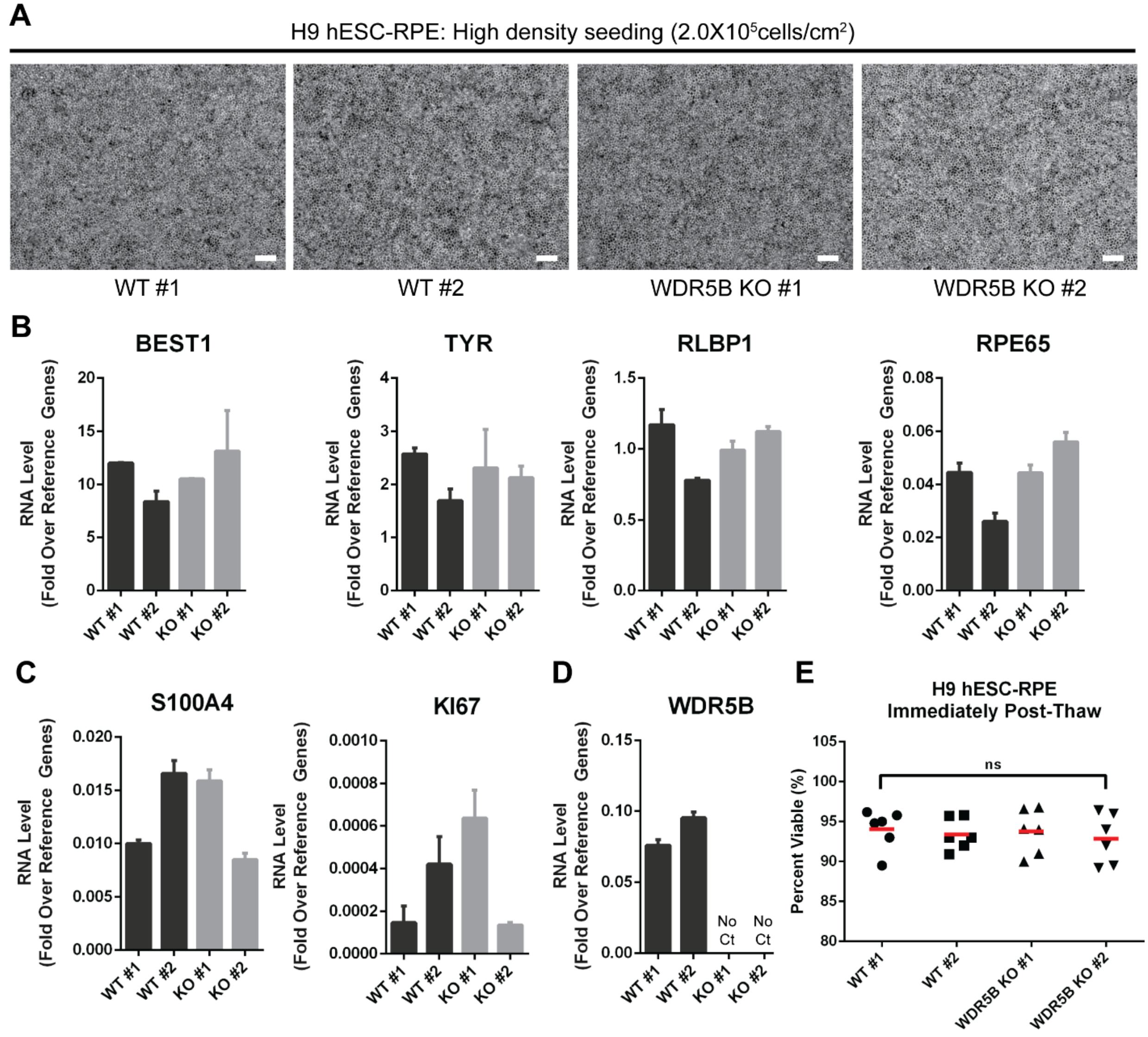
A
H9 hESC-RPE: High density seeding (2.0X10^5 cells/cm^2)
WT #1
WT #2
WDR5B KO #1
WDR5B KO #2
B
BEST1
TYR
RLBP1
RPE65
RNA Level (Fold Over Reference Genes)
WT #1
WT #2
KO #1
KO #2
C
S100A4
KI67
D
WDR5B
No Ct
No Ct
E
H9 hESC-RPE Immediately Post-Thaw
Percent Viable (%)
ns
WDR5B KO #1
WDR5B KO #2

**Figure S4.** WDR5B knockout hESC-RPE exhibit normal RPE morphology and expression of RPE marker genes when seeded at high density. (**A**) Both wild type and WDR5B null hESC-RPE lines exhibit similar cobblestone RPE morphology when seeded at confluence ($2.0X10^5$ cells/$cm^2$, images captured on day 54 post-seeding). Scale bars: 100 µm. (**B**) Expression of the RPE marker genes BEST1, TYR, RLBP1, and RPE65 did not significantly differ by genotype when cells were seeded at high density (n=3 experimental replicates). (**C**) Levels of epithelial-to-mesenchymal transition (S100A4) and cell proliferation (MKI67) markers were also not significantly different by genotype in cells seeded at high density (**D**) WDR5B mRNA was detected in both wild type clones while no amplification (No Ct) was observed in either knockout clone (n=3 experimental replicates for all RT-qPCR). (**E**) Immediately upon thaw of cryopreserved stocks, no significant differences in viability among RPE lines were observed (n=6 replicates from three independent thaws). Red bars indicate group means. Statistical analyses were conducted by univariate ANOVA with Tukey's post-hoc (ns, not significant). Error bars are ±S.D.

**Table S1: Oligonucleotide sequences**

| ID | Sequence (5' to 3') or Catalog Number | Assay |
|---|---|---|
| WDR5B Forward | GCTCGTTGATGACGATAACCCTCCTGTCT | RT-qPCR |
| WDR5B Reverse | GGCACCTGCCTCTGCTATAATCCCATAGTT | RT-qPCR |
| WDR5 Forward | CCCGAACGGCAAATACATCCTG | RT-qPCR |
| WDR5 Reverse | TTCTTGTGGCCAGTGTACGTCTTC | RT-qPCR |
| EIF2B2 Forward | CACTCTGGCACTGGCAGCAAA | RT-qPCR |
| EIF2B2 Reverse | GGACTTCTTCAGGAGCCACAAACTTATGA | RT-qPCR |
| UBE2R2 Forward | CCCCTATTCACCACCTACCTTCAGAT | RT-qPCR |
| UBE2R2 Reverse | CTACAGGCGGATGAAGAATCGAAATGC | RT-qPCR |
| EIF2B2 | Hs00204540_m1 (Thermo Fisher Scientific) | Taqman RT-qPCR |
| UBE2R2 | Hs00215107_m1 (Thermo Fisher Scientific) | Taqman RT-qPCR |
| RPE65 | Hs01071462_m1 (Thermo Fisher Scientific) | Taqman RT-qPCR |
| S100A4 | Hs00243202_m1 (Thermo Fisher Scientific) | Taqman RT-qPCR |
| BEST1 | Hs00188249_m1 (Thermo Fisher Scientific) | Taqman RT-qPCR |
| RLBP1 | Hs00165632_m1 (Thermo Fisher Scientific) | Taqman RT-qPCR |
| TYR | Hs00165976_m1 (Thermo Fisher Scientific) | Taqman RT-qPCR |
| MKI67 | Hs01032443_m1 (Thermo Fisher Scientific) | Taqman RT-qPCR |
| PUM1 Forward | TATGAAGGGACAATCTGCTC | CUT&RUN -qPCR |
| PUM1 Reverse | AATCCATCTTCATCCTACCG | CUT&RUN -qPCR |
| RPS6 Forward | GAGACCCTTCTCCACCTAAA | CUT&RUN -qPCR |
| RPS6 Reverse | CGAGTGTTAGACTGGGTTTG | CUT&RUN -qPCR |
| RPL26 Forward | GGTCTGGAAATTCCGTGAAGACTTT | CUT&RUN -qPCR |

| | | |
|---|---|---|
| RPL26 Reverse | GTCTGGGTAGTGTCGTTTCCTAGAG | CUT&RUN-qPCR |
| Negative Control Forward | AGGGCTATAAATTTACCTTTAAGTTCAGCT | CUT&RUN-qPCR |
| Negative Control Reverse | CCCCTCTGTTGAAAGCACAATACTT | CUT&RUN-qPCR |
| Nucleosome Forward | CGTATCGCGCGCATAATA | qPCR |
| Nucleosome Reverse | CGCGTAACGACGTACC | qPCR |
| Nucleosome Probe | /56-FAM/<br>TCTAGCACC/ZEN/GCTTAAACGCACGTA<br>/3IABkFQ/ | qPCR |
| WDR5B-3XFLAG HDR Template | C*T*C*ATCGCATCAGCAGCATTAGAAAATGACAAAACAATTAAACTGTGGATGAGTAACCACGATTACAAGGATGACGACGATAAGGGAGATTACAAGGATGACGACGATAAGATCGATTACAAGGATGACGACGATAAGTAATCCCTTTGAAAATCAAGTAGTAGAGCCAAGTTGGCCAAAAAACTTGGATATTTAAA*A*A*G | Knock-in HDR template |
| CD.Cas9.TFZZ6738.AD | /ALTR1/rUrUrGrArUrUrUrCrArArArGrGrGrArUrUrArGrGrUrUrUrUrArGrArGrCrUrArUrGrCrU/ALTR2/ | Knock-in crRNA |
| CD.Cas9.PVWS8678.AA | /ALTR1/rUrCrCrArArGrUrUrArGrCrArGrGrUrGrUrArCrUrGrUrUrUrUrArGrArGrCrUrArUrGrCrU/ALTR2/ | Knockout crRNA #1 |
| CD.Cas9.DJVX5152.AA | /ALTR1/rArUrCrArArGrUrArGrUrArGrArGrCrCrArArGrUrGrUrUrUrUrArGrArGrCrUrArUrGrCrU/ALTR2/ | Knockout crRNA #2 |
| F1 | CAAACTGCCAAAATTAAAAGAAACCGT | Knockout and Knock-in genotyping primer |
| R1 | ATCGCATCAGCAGCATTAGAAAAT | Knockout and Knock-in genotyping primer |
| R2 | GCTTTCAAAACTGTACATTTTAAAATCCTGTG | Knockout genotyping primer |
| WDR5B-FLAG Probe | /56-FAM/<br>CACGATTACAAGGATGACGACGATAAGGGAGA<br>/3IABkFQ/ | Knock-in genotyping probe |
| WDR5B-WT-Probe | /5HEX/<br>CCTTTGAAAATCAAGTAGTAGAGCCAAGTTGGCC<br>/3IABkFQ/ | Knockout and Knock-in |

| | | |
|---|---|---|
| | | genotyping probe |
| WDR5B-KO-Probe | /56-FAM/ CGTTCAGCC/ZEN/CTGAACCAGGCAGTCCAGTAC /3IABkFQ/ | Knockout genotyping probe |
| R3 | CATCATACCGGTGAAGTCCAAGCCGTGCTGCTG | Cloning |
| F2 | GATTGGTAGTCAGAAGCTGAATTCTAGATGTGC | Cloning |
| WDR5-FLAG Cloning XhoI Forward | CATCATCTCGAGGCCACCATGGCGACGGAGGAGAAGAAGC | Cloning |
| WDR5-FLAG Cloning NotI Reverse | TACTACGCGGCCGCTTACTTATCGTCGTCATCCTTGTAATCGCAGTCACTCTTCCACAGTTTAATTGTTTTGTC | Cloning |
| WDR5B-3XFLAG Cloning XhoI Forward | CATCATCTCGAGGCCACCATGGCAACCAAGGAGTCAAGAGAC | Cloning |
| WDR5B-3XFLAG Cloning XbaI Reverse | CATCATTCTAGATTACTTATCGTCGTCATCCTTGTAATCGATCTTATCG | Cloning |
| PLVX 2A Cloning XhoI Forward | CATCATCTCGAGGGAGATAGCGGATC | Cloning |
| PLVX 2A Cloning AgeI Reverse | GTAGTAACCGGTGGATGTGGAATGTGT | Cloning |
| WDR5B-NLUC-HA XbaI Forward | CATCATTCTAGAAATAATTTTGTTTAACTTTAAGAAGGAGATATACATATGGCAACCAAGGAGTCAAGAGAC | Cloning |
| WDR5B-NLUC-HA XhoI Reverse | CATCATCTCGAGGTGGTTACTCATCCACAGTTTAATTGTTTTGTC | Cloning |
| WDR5-NLUC-HA XbaI Forward | CATCATTCTAGAAATAATTTTGTTTAACTTTAAGAAGGAGATATACATATGGCGACGGAGGAGAAGAAG | Cloning |
| WDR5-NLUC-HA XhoI Reverse | CATCATCTCGAGGCAGTCACTCTTCCACAGTTTAATTGTTTTG | Cloning |

| | | |
|---|---|---|
| NLUC-HA XhoI Forward | CATCATCTCGAGGGCAGCAGCGGCATGGTCTTCACA CTCGAAGATTTCGTTG | Cloning |
| NLUC-HA BamHI Reverse | CATCATGGATCCTTAAGCGTAATCTGGAACATCGTAT GGGTAC | Cloning |
| NLUC-HA NdeI Forward | CATCATCATATGGTCTTCACACTCGAAGATTTCGTTG G | Cloning |
| NLUC-HA BlpI Reverse | CATCATGCTCAGCTTAAGCGTAATCTGGAACATCGT ATGGGTAC | Cloning |
| WDR5-FLAG Template | CATCATCTCGAGGCCACCATGGCGACGGAGGAGAA GAAGCCCGAGACCGAGGCCGCCAGAGCACAGCCAA CCCCTTCGTCATCCGCCACTCAGAGCAAGCCTACAC CTGTGAAGCCAAACTATGCTCTAAAGTTCACCCTTG CTGGCCACACCAAAGCAGTGTCCTCCGTGAAATTCA GCCCGAATGGAGAGTGGCTGGCAAGTTCATCTGCT GATAAACTTATTAAAATTTGGGGCGCGTATGATGGG AAATTTGAGAAAACCATATCTGGTCACAAGCTGGGA ATATCCGATGTAGCCTGGTCGTCAGATTCTAACCTTC TTGTTTCTGCCTCAGATGACAAAACCTTGAAGATATG GGACGTGAGCTCGGGCAAGTGTCTGAAAACCCTGA AGGGACACAGTAATTATGTCTTTTGCTGCAACTTCAA TCCCCAGTCCAACCTTATTGTCTCAGGATCCTTTGAC GAAAGCGTGAGGATATGGGATGTGAAAACAGGGAA GTGCCTCAAGACTTTGCCAGCTCACTCGGATCCAGT CTCGGCCGTTCATTTTAATCGTGATGGATCCTTGATA GTTTCAAGTAGCTATGATGGTCTCTGTCGCATCTGG GACACCGCCTCAGGCCAGTGCCTGAAGACGCTCAT CGATGACGACAACCCCCCCGTGTCTTTTGTGAAGTT CTCCCCGAACGGCAAATACATCCTGGCCGCCACGC TGGACAACACTCTGAAGCTCTGGGACTACAGCAAGG GGAAGTGCCTGAAGACGTACACTGGCCACAAGAAT GAGAAATACTGCATATTTGCCAATTTCTCTGTTACTG GTGGGAAGTGGATTGTGTCTGGCTCAGAGGATAACC TTGTTTACATCTGGAACCTTCAGACGAAAGAGATTGT ACAGAAACTACAAGGCCACACAGATGTCGTGATCTC AACAGCTTGTCACCCAACAGAAAACATCATCGCCTC TGCTGCGCTAGAAAATGACAAAACAATTAAACTGTG GAAGAGTGACTGCGATTACAAGGATGACGACGATAA GTAAGACTCTAGATAATTCTAC | Cloning |
| PLVXpuro P2A T2A Template | CATCATCTCGAGGGAGATAGCGGATCCGGCAGCGG CGCCACAAACTTCTCTCTGCTAAAGCAAGCAGGTGA TGTTGAAGAAAACCCAGGGCCTGAATTCGCTGGAAA GCGCGGCCGCGGCTCCGGCGAGGGCAGGGGAAGT | Cloning |

| | | |
|---|---|---|
| | CTTCTAACATGCGGGGACGTGGAGGAAAATCCCGG CCCACCCGGGGGAGCATCCGGACTCTAGATAATTCT ACCGGGTAGGGGAGGCGCTTTTCCCAAGGCAGTCT GGAGCATGCGCTTTAGCAGCCCCGCTGGGCACTTG GCGCTACACAAGTGGCCTCTGGCCTCGCACACATTC CACATCCACCGGTTACTAC | |
| NLUC-HA Template | ATGGTCTTCACACTCGAAGATTTCGTTGGGGACTGG CGACAGACAGCCGGCTACAACCTGGACCAAGTCCTT GAACAGGGAGGTGTGTCCAGTTTGTTTCAGAATCTC GGGGTGTCCGTAACTCCGATCCAAAGGATTGTCCTG AGCGGTGAAAATGGGCTGAAGATCGACATCCATGTC ATCATCCCGTATGAAGGTCTGAGCGGCGACCAAATG GGCCAGATCGAAAAAATTTTTAAGGTGGTGTACCCT GTGGATGATCATCACTTTAAGGTGATCCTGCACTAT GGCACACTGGTAATCGACGGGGTTACGCCGAACAT GATCGACTATTTCGGACGGCCGTATGAAGGCATCGC CGTGTTCGACGGCAAAAAGATCACTGTAACAGGGAC CCTGTGGAACGGCAACAAAATTATCGACGAGCGCCT GATCAACCCCGACGGCTCCCTGCTGTTCCGAGTAAC CATCAACGGAGTGACCGGCTGGCGGCTGTGCGAAC GCATTCTGGCGTACCCATACGATGTTCCAGATTACG CTTAA | Cloning |
| Control shRNA | CAACAAGATGAAGAGCACCAA (Sigma Aldrich #SHC002) | shRNA |
| WDR5B shRNA #1 | CCTTATAATCTCGGGATCTTT (Sigma Aldrich #TRCN0000117883) | shRNA |
| WDR5B shRNA #2 | GCAACTTTGGACAACACTCTT (Sigma Aldrich #TRCN0000117884) | shRNA |

**Modification Notes:**
***** Indicates a phosphorothioate bond
**r** Indicates an RNA base
**ALTR1 and ALTR2** Indicate crRNA-tracrRNA hybridization sequences
/**56-FAM**/ indicates a 5' 6-FAM (fluorescein) fluorophore
/**5HEX**/ indicates a 5’ hexachlorofluorescein fluorophore
/**ZEN**/ indicates the IDT ZEN internal fluorescence quencher
/**3IABkFQ**/ indicates the IDT 3' Iowa Black FQ fluorescence quencher
